# Formalizing digital health competencies for the health workforce in Ukraine

**DOI:** 10.3389/fpubh.2026.1842069

**Published:** 2026-05-21

**Authors:** Oleksandr Zvinchuk, Liubov Sergeeva, Tatiana Nanaieva, Vadym Terentyuk, Hlib Aleksandrenko

**Affiliations:** 1 Zhytomyr Medical Institute of Zhytomyr Oblast State Administration, Zhytomyr, Ukraine; 2 USAID Health Reform Support Project, Kyiv, Ukraine; 3 Ministry of Education and Science in Ukraine, Kyiv, Ukraine; 4 Bogomolets National Medical University, Kyiv, Ukraine; 5 School of Public Health of the National University of Kyiv-Mohyla Academy, Kyiv, Ukraine

**Keywords:** competency framework, digital health competencies, digital literacy, eHealth, health policy, health professional education, health workforce, Ukraine

## Abstract

Effective digital health transformation requires a workforce with adequate digital competencies, yet fewer than half of WHO European Region countries have policies addressing this gap. In Ukraine, a 2021 assessment found that 56% of healthcare professionals demonstrated only basic or beginner levels of digital literacy, while no standardized competency requirements existed across qualifications or education. The national Digital Competence Framework for Healthcare Professionals was developed through coordination among health, education, and digital authorities. The Framework defines 25 components across five domains—general digital literacy, health data management, digital communication, digital health tools, and digital transformation leadership—with descriptors at four proficiency levels, informing qualification standards, curricula, and competence assessment. This Ukrainian experience offers practical insights for countries seeking to formalize digital competencies for the health workforce.

## 1 Introduction

Digital health technologies are reshaping healthcare systems worldwide. The WHO Global Strategy on Digital Health 2020–2027 emphasizes that effective digital transformation depends on a health workforce equipped with appropriate digital competencies (1). This priority is further reinforced by the WHO Regional Digital Health Action Plan 2023–2030 for the European Region, which calls on Member States to strengthen digital literacy and capacity building for the health workforce (2). However, implementation has not kept pace with policy ambition. Fewer than half of the 53 Member States in the WHO European Region have adopted policies specifically aimed at improving digital health literacy among health professionals (3).

Several developed countries and regions have responded by developing competency frameworks. In Europe, the WHO-ASPHER Competency Framework for the Public Health Workforce provides a reference structure for public health roles, and the ASPHER Task Force on Digital Transformation has called for integrating digital competencies across public health curricula rather than in standalone courses (4, 5). National frameworks have also emerged in high-income settings: the Australian Digital Health Capability Framework covers the entire healthcare workforce across five domains and three capability levels (6); the Canadian Nursing Informatics Entry-to-Practice Competencies focus on registered nurses (7); the AAMC Telehealth Competencies target physicians at three developmental stages (8); and the regional COMPDIG-Salut framework in Catalonia integrates competence definition with assessment and accreditation (9). A 2020 scoping review of 30 educational frameworks found them concentrated in high-income countries, often targeting a single professional group, and rarely connected to regulatory instruments (10). A recent WHO initiative reached a similar conclusion: existing frameworks remain fragmented and narrowly focused (11). A mapping of digital public health training across five European countries similarly found initiatives expanding rapidly but fragmented, dominated by short formats, and insufficiently integrated into academic curricula (12). Low- and middle-income countries, and conflict-affected settings in particular, remain underrepresented.

Ukraine illustrates both the opportunities and challenges of rapid digital health transformation. The country has built a national digital health ecosystem (eHealth), with the Electronic Health System (EHS) as its core component (13). Since 2017, the EHS has registered more than 32 million patients, over 390,000 healthcare professionals, and more than 6,000 healthcare facilities (14). It enables electronic medical records, e-prescriptions, e-referrals, and patient portals. Ukraine has also developed multiple specialized national digital tools, information systems, and registers (15). However, the expansion of digital infrastructure has outpaced workforce preparedness, creating a growing gap between system capability and high-quality use in practice.

A national assessment conducted in 2021 across 2,554 healthcare professionals from 98 facilities in three regions of Ukraine revealed significant gaps in digital skills. Only 44% demonstrated above-basic digital literacy, while 35% had basic skills, and 21% remained at a beginner level. Lower literacy correlated with older age, rural practice, nursing roles, and lack of recent training. Only 37% had received digital literacy training in the preceding 3 years (16).

These gaps translate into operational challenges, further intensified by the full-scale war against Ukraine since February 2022, which has strained the workforce, heightened the importance of cybersecurity, and accelerated reliance on digital health tools. An estimated 40% of healthcare professionals reported not fully understanding the principles of secure electronic document management, while 55% indicated that medical information systems slowed their work rather than improving efficiency. A 2023 telemedicine assessment further highlighted limited readiness: 83% of health professionals had no training in telemedicine services, and 66% considered their telemedicine knowledge insufficient (17).

Despite these workforce needs, the regulatory framework for digital health competencies remained fragmented, providing no consistent basis for educational planning. Digital competency courses accounted for only one to 6% of continuing professional development (CPD) offerings. Digital skills requirements were also unevenly reflected across qualification standards, certification processes, and continuing education programs. The Handbook of Qualification Characteristics for Healthcare Professions (Issue 78) describes over 250 professional positions, yet defines no standardized digital competency requirements (18).

This paper introduces Ukraine’s Digital Competence Framework for Healthcare Professionals as a national policy instrument that harmonizes digital competency expectations, standardizes digital health education, and guides capacity-building. The Ukrainian experience contributes a whole-workforce, regulatorily anchored framework from a low- and middle-income, conflict-affected setting.

## 2 Policy options and implications

### 2.1 Scope and intended audience

The Digital Competence Framework for Healthcare Professionals (hereinafter—the Framework) applies to all professional groups in the healthcare sector. It addresses practicing professionals at all career stages, from students in medical education to experienced practitioners and managers, as well as educators developing training programs, and regulators updating qualification standards. The Framework is not designed for IT specialists working in healthcare, whose competencies fall within a separate professional domain.

### 2.2 Regulatory and policy foundations

Cabinet of Ministers of Ukraine Order no 1671-р (December 28, 2020) approved the Concept for eHealth Development, positioning digital transformation as a strategic priority for the health sector (23). Order no 167-р (March 3, 2021) approved the Concept for Digital Competencies Development, mandating the development of competency frameworks across sectors (22). These commitments were further operationalized through the National Strategy for Creating a Barrier-Free Space in Ukraine until 2030, which tasked the Ministry of Health with developing a digital competence framework for healthcare professionals (21).

Before 2023, Ukraine had already developed digital competency frameworks for citizens, entrepreneurs, civil servants, and educators (DigCompUA) (19). However, no equivalent framework existed for the health workforce.

### 2.3 Development process

The development of the Framework was led by intersectoral coordination among the Ministry of Health (MOH), the Ministry of Digital Transformation (MDT), and the Ministry of Education and Science (MES), with technical support from the USAID Health Reform Support (USAID HRS) project. In September 2023, an intersectoral working group was formally established by MOH Order No. 1627 (20). Chaired by the Deputy Minister of Health for Digital Development, the group included representatives from MOH (Department of Digital Transformation; Division of Medical Personnel, Education and Science), MES (Directorate of Higher Education), MDT (Directorate of European Integration), and the USAID HRS project.

The Framework was informed by both national policy priorities and international conceptual models. National foundations included the National Strategy for Creating a Barrier-Free Space in Ukraine until 2030 (21), the Concept for the Development of Digital Competencies (22), and the Concept for the Development of Electronic Healthcare (23). International foundations included the European Union’s DigComp 2.1 and DigComp 2.2 frameworks (31), the Ukrainian Digital Competence Framework for Citizens (19), and digital health competency approaches from countries with advanced digital health systems: the UK’s patient-centered eHealth competency approach (24), US and Canadian role-based health informatics frameworks (25, 26), Israel’s innovation-driven digital health model (27), and Singapore’s WHO Collaborating Centre in Digital Health and Education (28). Germany (29) and Estonia (30) informed competencies on telemedicine, artificial intelligence, clinical decision support, data analytics, cybersecurity, and data protection. These sources helped identify common global domains and adapt them to Ukraine’s eHealth context.

Benchmarking of international frameworks identified recurring domains relevant to digital health practice, including digital literacy, health data management, digital communication, ethical and legal standards, cybersecurity, and the clinical use of digital technologies. These domains were adapted to EHS, clinical workflows, medical information systems, telemedicine, medical devices, artificial intelligence, and the functional responsibilities of healthcare professions.

The Framework translates general digital competence concepts into a specialized healthcare model and reflects the European Qualifications Framework (EQF) (33). It simplifies the eight DigComp proficiency levels into four practical levels for Ukraine’s health workforce: Basic, Sufficient, Professional, and High. These levels are structured around task complexity, autonomy, and cognitive domain, aligned with Bloom’s taxonomy progressing from remembering and understanding to application, evaluation, critical thinking, innovation, and creative implementation. The Framework includes a dedicated domain on Digital Transformation in Healthcare, addressing digital leadership, workflow automation, business analytics, and organizational cyber risk management. This domain responds to a gap in many international models, which often focus on clinical and technical competencies but provide limited guidance on institutional digital transformation.

The Framework was refined through extensive intersectoral consultation that functioned as a structured expert dialogue. The draft was reviewed with representatives of pre-higher, higher, and postgraduate medical and pharmaceutical education institutions, healthcare facilities, Medical Information System developers, IT specialists, and national authorities. The development timeline included expert consultation on initial drafts (March–April 2023), a public consultation period (May 2023), formal establishment of the intersectoral working group (September 2023), and final approval of the Framework (October 6, 2023). Following approval, the Framework was published on the MOH website and disseminated to educational institutions nationwide (34). An online launch event attracted over 2,000 participants from healthcare facilities, medical and pharmaceutical education institutions, and regional health departments from 20 regions of Ukraine.

### 2.4 Framework structure

**Figure 1:**
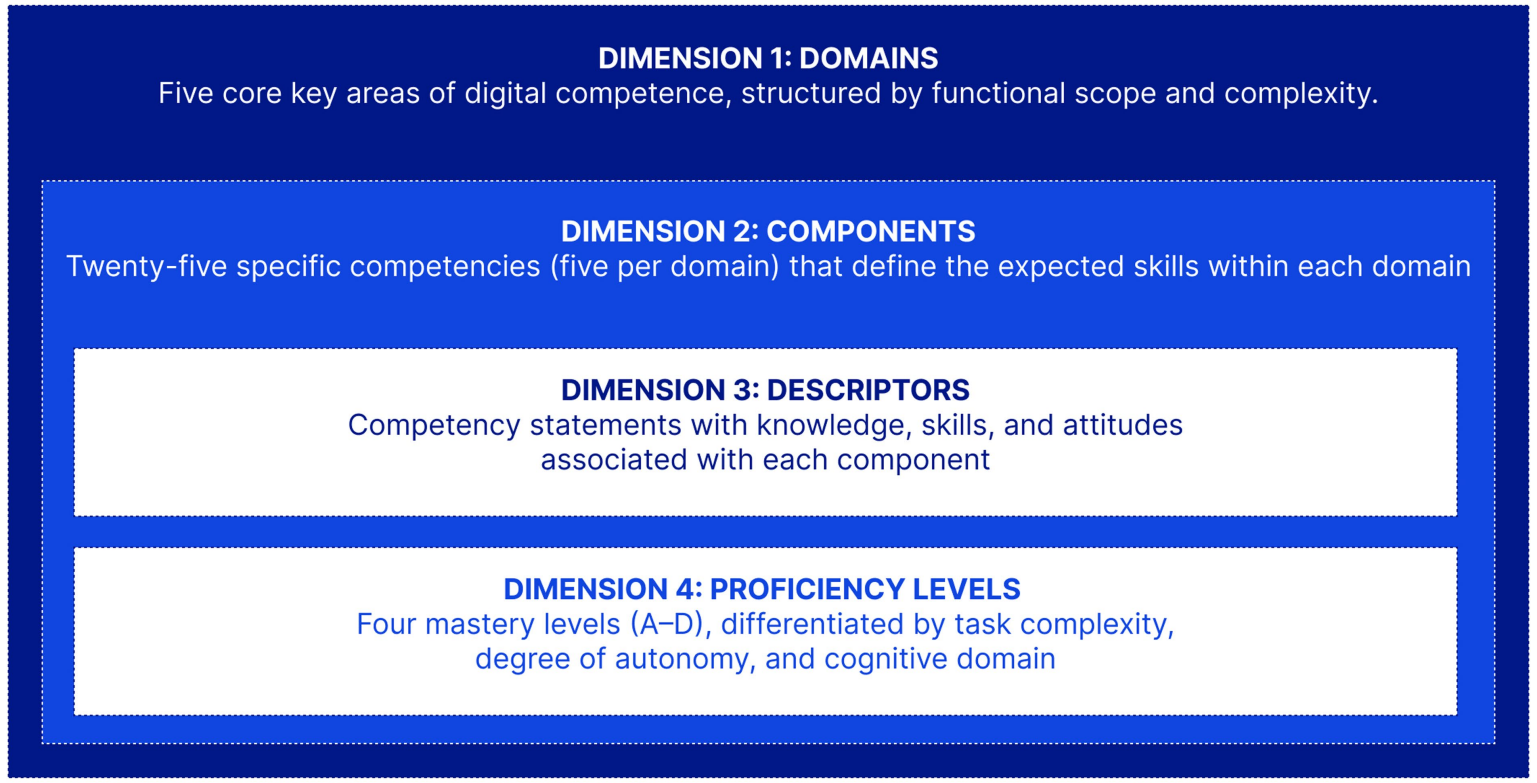
The four dimensions of the digital competence framework for healthcare professionals: domains, components, descriptors, and proficiency levels.

The Framework organizes digital competencies across four dimensions, which together provide a structured basis for defining, teaching, and assessing digital skills among healthcare professionals. Figure 1 summarizes these dimensions.

Together, these four dimensions form the operational architecture of the Framework. The complete content, all 25 components with their descriptors and proficiency-level descriptions, is provided in Supplementary Table S1.

*Dimension 1: Domains*. The Framework specifies five domains that capture distinct areas of digital competence required of healthcare professionals. Domain 1 (General Digital Literacy) establishes foundational competencies relevant across all healthcare roles. Domains 2–4 focus on patient-centered digital practice, covering health data management, digital communication, and the use of advanced technologies. Domain 5 addresses leadership and management competencies needed to plan, implement, and sustain digital transformation in healthcare settings.

**Figure 2:**
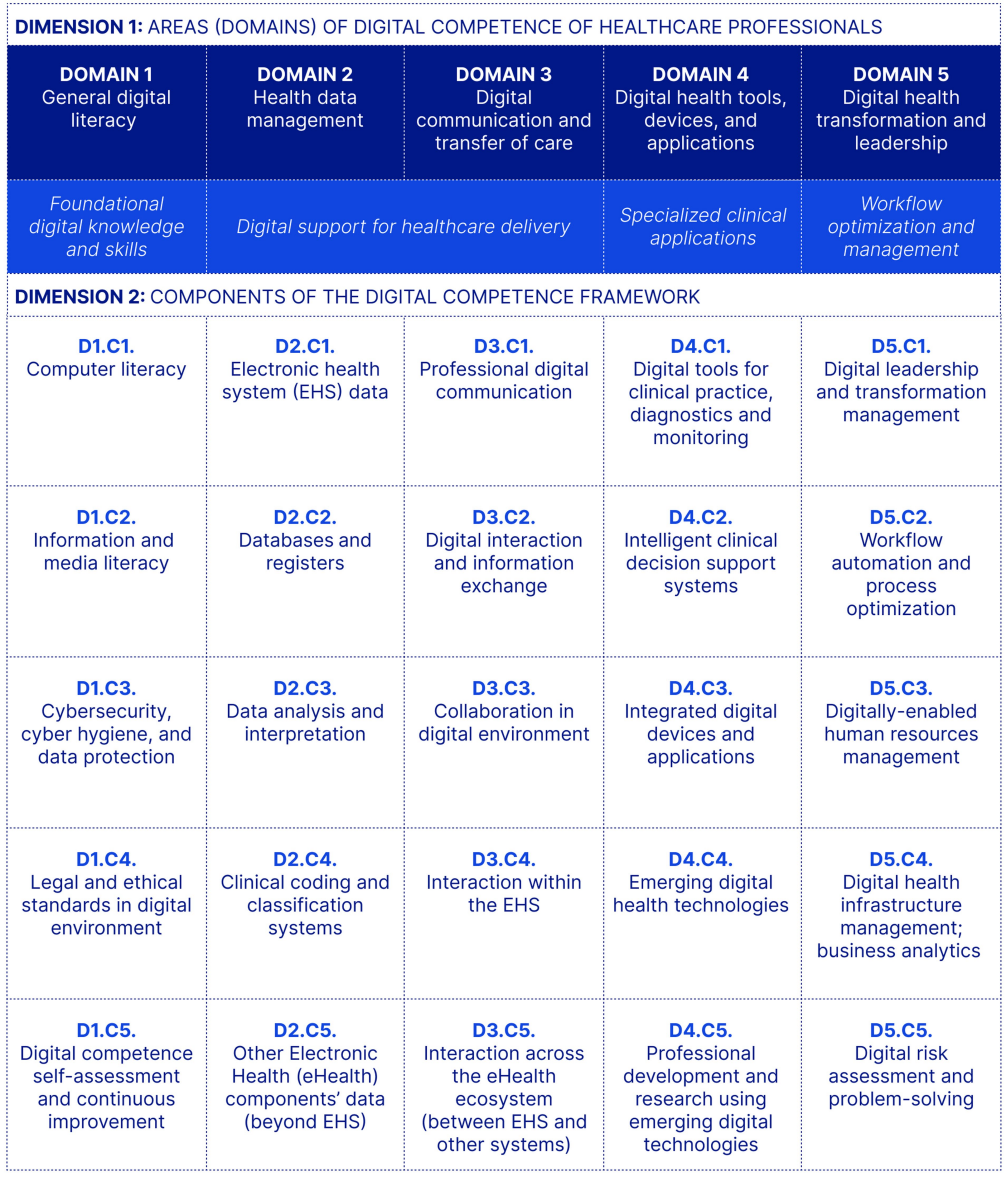
The five domains of the framework and their 25 components. The notation Dx.Cy denotes Domain x, Component y.

*Dimension 2: Components*. Each domain is divided into five components, resulting in 25 components overall. These components operationalize the domains by defining the specific competencies expected within each area (Figure 2).

**Figure 3:**
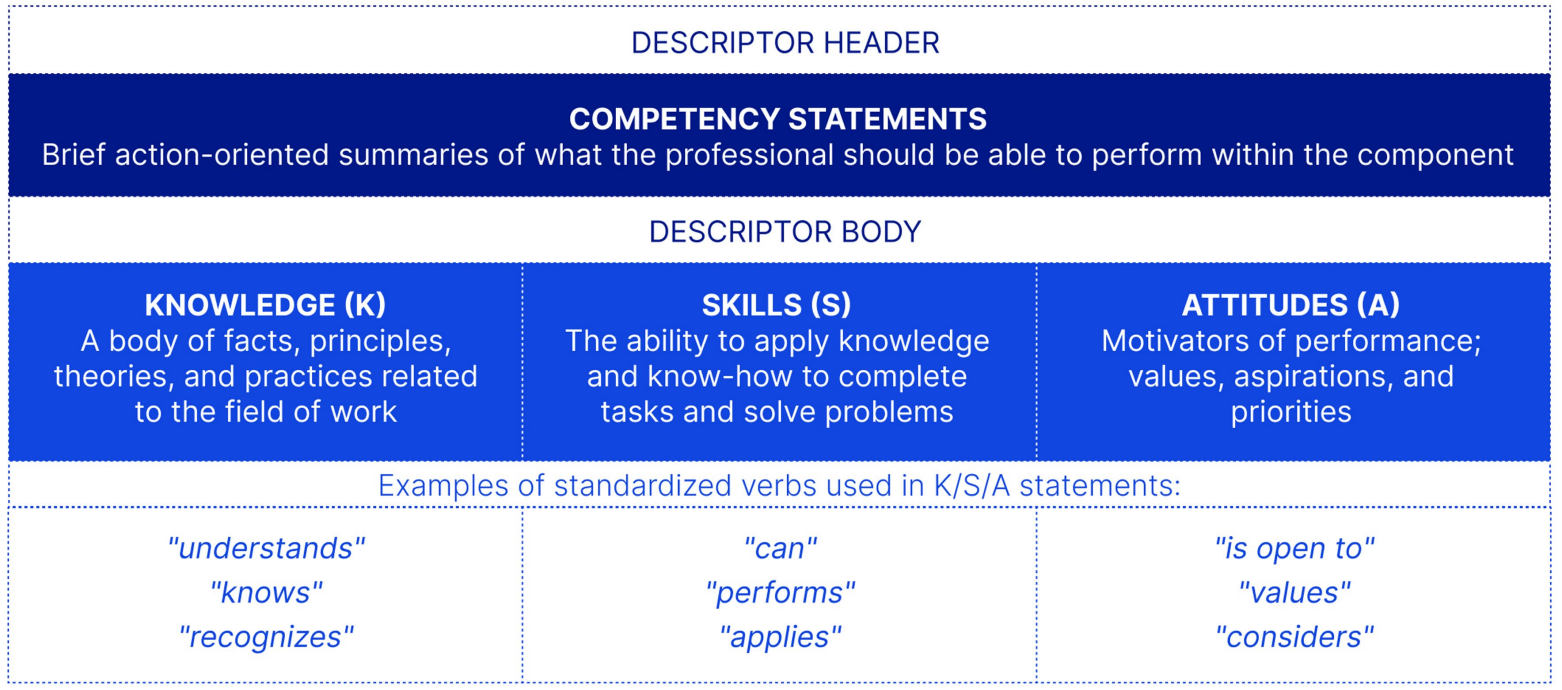
The structure of a component descriptor. Each descriptor includes competency statements specifying required knowledge (K), skills (S), and attitudes (A) using standardized verb formulations. The header–body grouping is illustrative.

*Dimension 3: Descriptors*. Each component is elaborated through a descriptor that includes multiple competency statements—brief action-oriented summaries of what the professional should be able to perform—and specifies the required knowledge (K), skills (S), and attitudes (A) using standardized verb formulations (Figure 3). Beyond this structure, descriptors serve as the Framework’s operational instruments: they guide the design of training modules and assessments, and provide the basis for formulating digital competence requirements across qualification standards.

**Figure 4:**
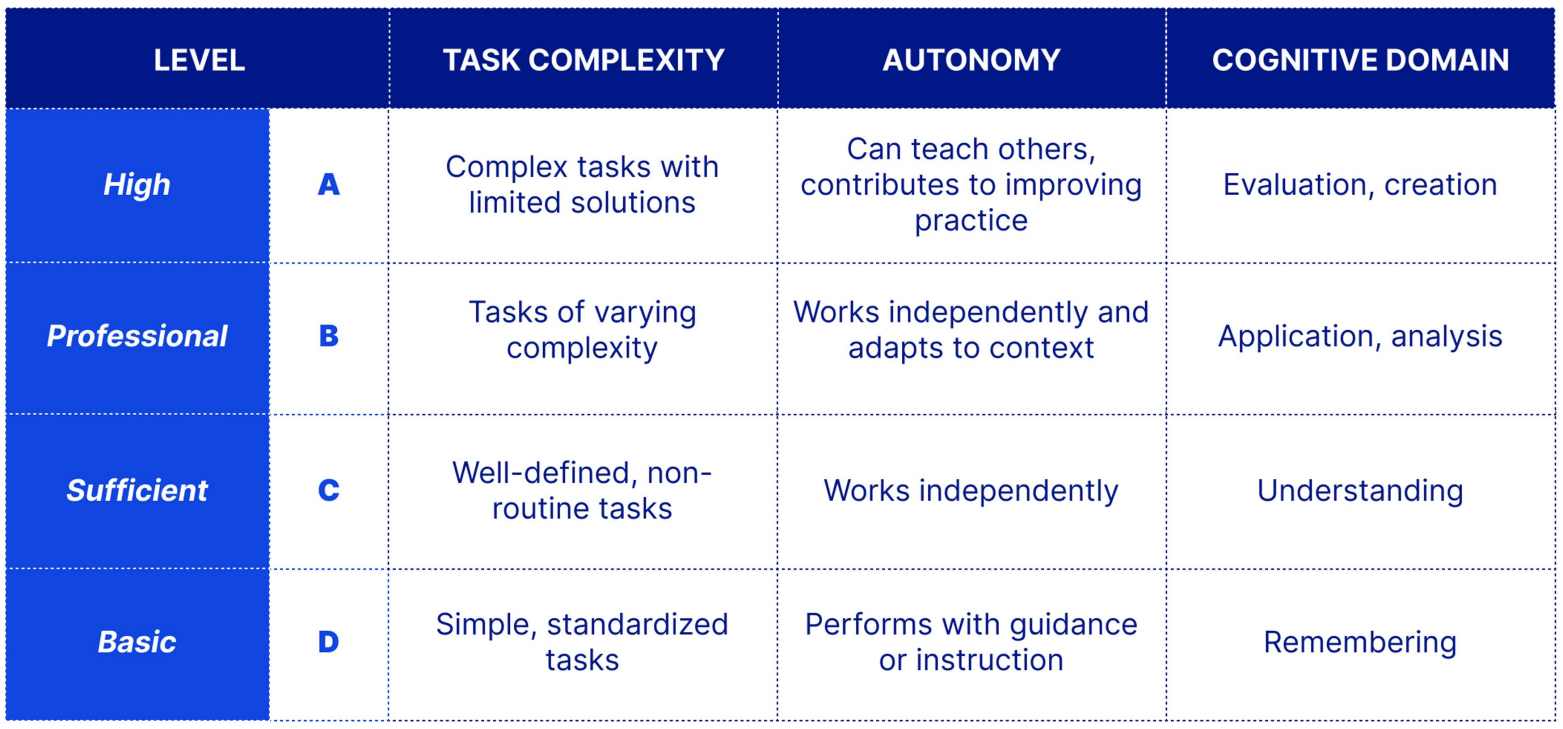
The four proficiency levels (A–D) of the framework, differentiated by task complexity, degree of autonomy, and cognitive domain. Each higher level subsumes the competencies of previous levels.

*Dimension 4: Proficiency Levels*. The Framework defines four proficiency levels (A through D) that describe progression from basic to expert competence. Each level is differentiated using three parameters: task complexity, degree of autonomy, and cognitive domain required to perform the task. Figure 4 summarizes the structure of the proficiency levels.

Each higher level subsumes the competencies of previous levels, reflecting a cumulative progression in capability. Expected proficiency may therefore differ across professional roles, career stages, scopes of practice, and organizational contexts. Rather than assigning fixed levels to specific positions, the Framework serves as a reference model for qualification standards, and educational programs can adapt and apply them in line with their respective requirements.

### 2.5 Framework applications and piloting

The Framework document outlines five application pathways: (I) policy development, to inform national digital health strategies and regulatory standards; (II) professional standards, to support updates to qualification characteristics and certification requirements; (III) educational programs, to guide curriculum design in undergraduate, graduate, and continuing professional development tracks; (IV) organizational planning, to inform workforce development planning at healthcare facilities; and (V) assessment tools, to underpin self-assessment and competency evaluation instruments.

Initial implementation took place through a grant program at two medical education institutions: Bogomolets National Medical University and Zhytomyr Medical Institute, from November 2023 to August 2024. The grant program supported the development of 17 updated curricula and 21 specialized study guides across five healthcare specialties (35, 36). In 2024, 1,712 students enrolled in the updated programs, and 576 healthcare professionals completed updated continuing professional development tracks (35, 36). A complementary analysis of 23 curricula across 11 medical education institutions identified misalignments with the Framework and informed criteria for curriculum updates (37). Subsequent empirical evaluation of the updated curricula at Zhytomyr Medical Institute documented improvement in measured digital competence among nursing students and practicing nurses (38).

A digital competency self-assessment tool aligned with the Framework was developed and piloted with 664 medical professionals and students. The tool is launched on the national Diia.Osvita platform (39).

## 3 Actionable recommendations

The following recommendations reflect lessons learned during the development and early implementation of the Framework in Ukraine. They are grounded in the Framework’s distinguishing features—cross-sectoral coordination, regulatory anchoring, whole-workforce coverage, and development in a low- and middle-income, conflict-affected setting. They are offered as considerations rather than prescriptions, recognizing that adaptation to national context, system maturity, and regulatory environment is essential.

### 3.1 For countries considering framework development

Countries without standardized digital competency requirements for healthcare professionals should assess their policy landscape before initiating framework development. This assessment helps identify key stakeholders, ensures the framework is informed by existing policies and strategies such as national digital health strategies and interoperability initiatives, and supports long-term sustainability through alignment with health system priorities. For accession countries, alignment with the broader EU digital health agenda strengthens both the technical and political case for framework development.

Cross-sectoral leadership is critical, as digital competencies span clinical practice, educational standards, and technology policy. Designating a lead ministry, typically health, while establishing formal coordination with education and digital agencies prevents fragmented approaches and ensures regulatory coherence. Political commitment should be secured through formal policy instruments such as government action plans or ministerial orders, which create accountability and signal institutional priority. European public health scholarship similarly emphasizes that digital competencies should be embedded across curricula and institutional structures rather than developed as standalone or advisory documents (5).

Beyond government coordination, sustained engagement of education institutions, professional associations, healthcare providers, and development partners broadens the stakeholder base and supports adaptation to diverse institutional landscapes.

### 3.2 For framework design and adaptation

The four-dimensional architecture (domains, components, descriptors, and proficiency levels) provides a transferable structure that countries can adopt while adapting content to local contexts. The development process is equally transferable. Descriptor content, by contrast, is inherently context-specific and reflects each country’s digital health systems, clinical coding standards, and regulatory environment.

International benchmarks should inform, not constrain, framework design. DigComp 2.2 offers a validated conceptual foundation for general digital competencies, while healthcare-specific frameworks such as the UK Health and Care Digital Capabilities Framework (32) provide sector-relevant guidance. Direct adoption of foreign frameworks rarely succeeds; adaptation that preserves structural compatibility while addressing local health system characteristics yields more sustainable results. The recent international consensus on digital health competencies in medical education (DECODE) similarly emphasizes adaptation to national education and regulatory contexts as a precondition for effective uptake (40).

The Framework maps each competence area directly to functions of the national eHealth and to strategic documents on digital transformation and barrier-free access. Anchoring competencies in concrete system functions, rather than presenting them as a generic skills inventory, strengthens both technical fit and political support.

Framework design should balance comprehensiveness with practicality. Including advanced technology competencies (artificial intelligence, Internet of Medical Things, clinical decision support) prepares the workforce for emerging tools, while core competencies in health data management and digital communication remain foundational. Proficiency levels should be defined with sufficient clarity to guide educational assessment and professional certification.

### 3.3 For implementation planning

Integration into educational standards represents the primary implementation pathway. Medical education institutions should map existing curricula against framework components to identify gaps. Component descriptors provide ready-made learning objectives; proficiency levels offer benchmarks for assessment design. Given the limited time available within existing curricula, integration of digital competencies across clinical and public health disciplines may be more feasible than introducing standalone courses. Recent literature on integrating digital health into medical curricula identifies a consistent set of barriers, including curriculum density, lack of clinically trained faculty with technical expertise, fragmented governance of educational standards, and absence of standardization across institutions (41).

Qualification requirements and professional standards should reference framework competencies explicitly. Updating handbooks of qualification characteristics or professional certification criteria creates regulatory demand that drives educational response. Without formal linkage to professional standards, frameworks remain advisory documents with limited uptake. International experience indicates that linking digital competencies to professional accreditation, credentialing, and licensing standards is essential for sustained workforce-level implementation (42).

Assessment tools aligned with the Framework enable both individual self-assessment and organizational workforce planning. A recent scoping review of instruments to assess digital health competencies highlights the need for psychometrically validated tools mapped to structured competency frameworks (43). Self-assessment instruments help healthcare professionals identify development needs; facility-level assessments support training prioritization and could be integrated into digital maturity scoring. Implementation expectations should be calibrated to the maturity of the digital health infrastructure: more mature systems can sustain higher proficiency expectations, while less mature contexts may prioritize foundational competencies (11).

### 3.4 For international organizations

International organizations, particularly the WHO and regional bodies, can accelerate the adoption of frameworks by promoting structural harmonization across countries. Aligned dimensional architectures facilitate cross-national comparison, support health workforce mobility, and enable benchmarking of digital competency levels.

Technical assistance for countries initiating framework development should emphasize adaptation methodology rather than template transfer. Supporting stakeholder engagement processes, providing access to international benchmarks, and facilitating peer learning among countries at different implementation stages adds more value than prescriptive models.

Documentation and dissemination of implementation outcomes remain limited globally. International organizations can address this gap by establishing repositories of framework documentation, commissioning comparative analyses, and convening knowledge exchange among implementing countries. As digital health technologies evolve rapidly, coordination on framework updates—particularly for emerging competencies in artificial intelligence and data analytics—helps prevent fragmentation and maintain cross-national compatibility.

## 4 Conclusion

Ukraine has developed a Digital Competence Framework for Healthcare Professionals through intersectoral coordination among the ministries responsible for health, digital transformation, and education. The Framework comprises five domains, 25 components with their descriptors, and four proficiency levels, providing a standardized reference for policy, education, and workforce development. Its distinguishing features—whole-workforce coverage, regulatory anchoring through qualification standards, and development in a low- and middle-income, conflict-affected setting—offer practical insights for other countries seeking to formalize digital competencies for the health workforce. Key next steps include evaluating implementation and learning outcomes, updating framework content as technologies and workflows evolve, and developing validated assessment tools that map directly to the defined competencies and proficiency levels.
