# Supplementary material for: Formalizing digital health competencies for the health workforce in Ukraine
Source: Front Public Health. 2026 May 21;14:1842069. doi: 10.3389/fpubh.2026.1842069 (PMC13233498; doi:10.3389/fpubh.2026.1842069)
Supplement: Supplementary file 1 [file Data_Sheet_1.docx]

**Supplementary Table S1.** Descriptors and proficiency-level descriptions for the 25 components of the Digital Competence Framework for Healthcare Professionals (Unofficial English translation of Appendix 1 of the Digital Competence Framework for Healthcare Professionals, Ministry of Health of Ukraine Order No. 1627, 14 September 2023). For each component across the five domains, the table presents the descriptor and the corresponding knowledge, skills, and attitudes, together with descriptions for four proficiency levels (A — Basic, B — Sufficient, C — Professional, D — High). The authoritative version is the original Ukrainian-language document, which is publicly available on the Ministry of Health of Ukraine website at [https://moz.gov.ua/uk/ramka-cifrovih-kompetentnostej-pracivnika-ohoroni-zdorov-ya.](https://moz.gov.ua/uk/ramka-cifrovih-kompetentnostej-pracivnika-ohoroni-zdorov-ya) In case of any discrepancy between this translation and the original, readers should refer to the original Ukrainian text, which prevails.

| **DOMAIN 1. GENERAL DIGITAL LITERACY** | |
| --- | --- |
| **D1.C1. COMPUTER LITERACY** | |
| **DESCRIPTOR** | |
| Use of modern means of informatization.  **Use of computer and mobile devices.** Configuring and applying computer and mobile devices for personal and work purposes, taking into account professional specialization and job responsibilities.  **Use of basic software.** Ability to apply standard software (word processors, graphic editors, spreadsheets, and other software) on both computers and mobile devices; ability to install and work with the most common operating systems, online services, files of various types, and applications.  **Use of applications and professional software.** Use of applications and professional software on both computers and mobile devices; ability to install, configure, and master new applications and software.  **Organization of the digital workplace.** Configuring peripheral input and output devices; connecting to wired and wireless computer networks. | |
| **Knowledge** | - Knows computer and mobile devices. - Knows basic software, including operating systems, files of various types, word processors, graphic editors, spreadsheets, and other application software. - Knows the most common online services. - Knows peripheral input and output devices. - Knows the principles of connecting to wired and wireless computer networks. |
| **Skills** | - Can configure and apply computer and mobile devices for personal and work purposes, taking into account professional specialization and job responsibilities. - Can work with the most common operating systems, files of various types, and applications. - Can use online services. - Can apply standard software (word processors, graphic editors, spreadsheets, and other software) on a range of computers and on mobile devices. - Can use and configure peripheral input and output devices, such as printers, scanners, and multifunction devices. - Can connect to wired and wireless computer networks and provide internet access as needed. - Can create a mobile hotspot for internet connectivity. |
| **Attitudes** | - Is open to mastering new devices, software, and applications. - Recognizes both the positive and negative consequences of using computer devices. |
| **PROFICIENCY LEVELS** | |
| **Level A (Basic)** | **At a basic level, with some autonomy following an instruction or template, or under the guidance of others, can:** apply computer and mobile devices for professional purposes; work with the most common operating systems, files of various types, and applications; use the most widespread online services; create and edit electronic text documents; use peripheral input and output devices; connect to wired and wireless computer networks and use internet access. |
| **Level B (Sufficient)** | **At a sufficient level, independently, in line with one's own needs and solving clearly defined standard problems, can:** perform basic configuration of computer and mobile devices for personal and work purposes; work with the most common operating systems, files of various types, and applications; use a range of online services; create, edit, and format electronic text, spreadsheet, and graphic documents; create basic and use standard formulas; create, edit, and save files in various formats; use peripheral input and output devices; perform initial configuration of a local Wi-Fi network and provide internet access; create a mobile hotspot for internet connectivity. |
| **Level C (Professional)** | **Independently, in line with one's own needs and the needs of others, solving non-standard problems, can:** configure and apply computer and mobile devices for personal and work purposes; as needed, work with various operating systems; create, edit, and save electronic documents in various formats; use standard formulas, calculate statistics, create charts and graphs, and import and export structured data in various formats; create, edit, and save graphic files in various formats; use and configure peripheral input and output devices; provide internet access as needed. |
| **Level D (High)** | **At a high professional or specialized level, including in complex contexts, can:** design solutions for complex problems with multiple interacting factors related to reviewing, searching, filtering, managing, analyzing, and assessing reliable data sources, information, and content for organizing, storing, and retrieving them in a structured digital environment; create digital content; and propose new ideas and processes in the field. |
| **D1.C2. INFORMATION LITERACY AND MEDIA LITERACY** | |
| **DESCRIPTOR** | |
| **Searching and filtering medical information, data, and digital content.** Knowledge of search engines and the ability to formulate information needs that meet professional duties and standards. Identification of trusted sources of medical information; recognition of credible sources within the Electronic Health System (EHS); ability to retrieve and use up-to-date data of confirmed reliability.  **Critical evaluation of information sources.** Ability to analyze, compare, and critically evaluate the credibility and reliability of medical sources, data, information, and digital content. Awareness of expert practices for working with information from various sources; awareness of evolving norms and ways of evaluating information.  **Management of medical information, data, and content.** Ability to organize, store, and retrieve data, information, and content in the EHS and Medical Information Systems (MIS); ability to operate them in a structured environment, including in cloud-based environments.  **Use of search and bibliographic systems.** Ability to use Ukrainian and international search and bibliographic services, scientometric databases (Scopus, Web of Science, PubMed, Google Scholar, and others) for professional development, research, and evidence-based practice. | |
| **Knowledge** | - Knows search engines, the most common online services, current norms for evaluating digital information, and rules for working with information from various sources. - Knows trusted sources of medical information, the architecture of the EHS, and the principles for organizing, storing, and retrieving data and content in the EHS and MIS. - Knows national and international search and bibliographic systems and scientometric databases. |
| **Skills** | - Can formulate information needs and articulate professional queries. - Can perform searches, filter results, and retrieve medical information from credible sources within and outside the EHS. - Can analyze, compare, and critically evaluate the credibility and reliability of medical information sources, data, and digital content. - Can organize, store, and retrieve data, information, and content in the EHS and MIS, including in cloud environments. - Can use Ukrainian and international search and bibliographic services and scientometric databases (Scopus, Web of Science, PubMed, Google Scholar, and others) for professional and research purposes. |
| **Attitudes** | - Values the credibility and reliability of medical information. - Is aware of the risks of misinformation and the importance of relying on evidence-based sources. - Considers the responsibility involved in handling, storing, and disseminating medical data. |
| **PROFICIENCY LEVELS** | |
| **Level A (Basic)** | **At a basic level, with some autonomy following an instruction or template, or under the guidance of others, can:** use common search engines and online services to retrieve medical information; identify trusted sources within the EHS; perform basic operations to organize, store, and retrieve data and content; use simple bibliographic searches in Ukrainian and international databases. |
| **Level B (Sufficient)** | **At a sufficient level, independently, in line with one's own needs and solving clearly defined standard problems, can:** formulate information queries; critically evaluate the credibility of sources; manage data, information, and content in the EHS and MIS; perform searches in scientometric databases (Scopus, Web of Science, PubMed, Google Scholar) and select sources relevant to professional tasks. |
| **Level C (Professional)** | **Independently, in line with one's own needs and the needs of others, solving non-standard problems, can:** perform advanced searches and filtering across credible sources; analyze and compare information from multiple databases; organize and structure complex sets of medical data and content in the EHS and MIS, including cloud-based environments; apply scientometric tools systematically for evidence-based practice. |
| **Level D (High)** | **At a high professional or specialized level, including in complex contexts, can:** design solutions to complex problems involving the review, retrieval, filtering, management, analysis, and evaluation of multiple data sources, information, and content; structure digital content for organization, storage, and retrieval in advanced digital environments; propose new ideas and processes for managing medical information. |
| **D1.C3. CYBERSECURITY, CYBER HYGIENE, AND DATA PROTECTION** | |
| **DESCRIPTOR** | |
| **Protection of computer devices and secure internet connection.** Understanding of risks and threats in digital environments. Knowledge of security and protection measures and appropriate consideration of reliability and privacy. Electronic identification — access control via biometric data, text passwords, tokens, smart cards, barcodes, PIN codes, and similar means. Adherence to the password policy of a specific organization or institution.  **Data protection.** Protection of personal data and privacy; use of passwords and encryption to protect files and data; security of data storage and cloud technologies; use of secure communication channels; adherence to safe use of mobile devices; internet security.  **Protection of health and well-being.** Ability to avoid risks and threats to physical and psychological health when using digital technologies. Ability to protect oneself and others from possible dangers in digital environments (for example, phishing). Adherence to ergonomic rules and to a balanced work–rest schedule to safeguard one's own health.  **Confidentiality and access control in the EHS.** Ability to apply key security principles within the EHS: identifying possible system vulnerabilities and adhering to formal organizational security policies at the personal, professional, and institutional levels. Ability to prevent the main threats to information leakage in MIS: incidental viewing, unauthorized requests, malicious damage, uncontrolled access, and risks associated with data transfer to external storage media. Implementation of measures to protect against threats to data security in MIS, including storage and backup. Understanding how to use and share personally identifiable information while protecting oneself and patients from harm. Use of systems with multi-factor authentication for the transfer of both personal and professional data. Knowledge of procedures to follow in the event of a cyber threat and the ability to apply them. | |
| **Knowledge** | - Knows the possible risks in the digital environment and the necessity of protecting computer devices. - Knows the principles of secure internet connection, electronic identification, access control, and security policies. - Knows methods of protecting personal data and privacy, including data protection on the internet and in cloud environments. - Knows how to safeguard personal health and well-being in the digital environment. - Knows the requirements for data protection and confidentiality when using EHS services. - Knows the procedures to follow during cyber threats and methods of avoiding them. - Knows the local security policy and is aware of computer and internet addiction risks. |
| **Skills** | - Can ensure the protection of computer devices. - Can establish a secure internet connection. - Can manage access control to confidential information, personal data, and online resources, including through biometric parameters, text passwords, tokens, smart cards, barcodes, and PIN codes. - Can use electronic digital signatures and qualified electronic signatures (including cloud-based) and tokens to access and submit data to state registries within the scope of authority. - Can avoid risks and threats to personal physical and psychological health and well-being when using digital technologies for personal needs or professional tasks. - Adheres to ergonomic and cyber hygiene rules and manages personal work–rest schedule when working with computer and professional digital equipment. - Can identify possible vulnerabilities of various components and software when working in the EHS. - Adheres to the basic principles, requirements, and security policy at the personal, professional, and institutional levels declared in a specific healthcare facility. - Prevents threats to information leakage when working with digital content in MIS: avoids incidental viewing, prevents unauthorized requests, malicious damage, uncontrolled access, and the risk of transferring restricted data to external storage media, including data leakage to the internet. - Implements data protection and security in MIS, including proper storage and backup. - Can apply scripts (action protocols) in cases of cyber threats. |
| **Attitudes** | - Pays attention to risks and dangers in the digital environment. - Is aware of personal responsibility for confidentiality and the protection of sensitive data, and of the consequences of failing to comply with these requirements. - Recognizes the importance of using data protection tools and technologies, particularly in MIS, and accepts responsibility for protecting data and personal devices within one's area of responsibility. - Independently of password policy requirements, creates passwords using all four character types (uppercase letter, lowercase letter, digit, special character) with a minimum length of eight characters. - Recognizes the necessity of multi-factor authentication for the transfer of both personal and professional data. - Prevents personal passwords from being visible or freely accessible to others; does not leave the computer unattended after authentication (logs out, locks the device, or exits the application before stepping away). - Adheres to the requirement of periodic password changes (for example, monthly, but no less than quarterly). - Adheres to the requirement of periodic data storage and backup checks. - Recognizes the necessity of installing and regularly updating antivirus software and firewalls on computer devices. - Complies with the requirements of a specific organization regarding security and password policies and access restrictions. |
| **PROFICIENCY LEVELS** | |
| **Level A (Basic)** | **At a basic level, with some autonomy following an instruction or template, or under the guidance of others, can:** work safely with computer tools and gadgets using password access, two-factor authentication, and other services recommended by a specific organization; ensure the protection of personal data and privacy through secure information transmission channels; avoid risks and threats to personal physical and psychological health and well-being and follow cyber hygiene standards; adhere to the basic principles, requirements, and security policy at the personal, professional, and institutional levels declared in a specific healthcare facility; prevent the leakage of confidential information when working with digital content in MIS and the EHS. |
| **Level B (Sufficient)** | **Independently, solving clearly defined and standard problems consistent with professional tasks, job responsibilities, and personal professional needs, can:** manage access control to confidential information, personal data, and online resources; protect files and data, ensure secure data transfer to and from storage and cloud resources, and ensure the safe use of mobile devices and online resources; use available services and resources to protect personal health and well-being; use software tools to prevent information leakage when working with digital content in MIS. |
| **Level C (Professional)** | **Independently, in line with one's own needs and the needs of others, solving non-standard problems, can:** use all available methods of electronic identification for protected and secure work in the digital environment, including adherence to the security policy of a specific organization; apply non-standard approaches to protecting personal and professional information and ensure the protection of data and digital tools and resources from unauthorized access at the workplace and in the network environment; apply non-standard approaches to protecting personal physical and psychological health and well-being; implement measures to protect against data security threats in MIS, including storage and backup; know and apply scripts (action protocols) in the event of a cyber threat. |
| **Level D (High)** | **At a high professional or specialized level, including in complex contexts, can:** with an understanding of risks and threats in digital environments, evaluate and propose various technologies and approaches for protecting computer devices and securing internet connectivity, and address problem-type tasks; propose own solutions and select the best options for protecting personal data, privacy, and personal well-being when using computer tools for personal needs or professional tasks; analyze and improve the security policy of a specific organization with regard to MIS and EHS resources; perform comparative analysis and select systems, tools, and resources that implement best practices in cybersecurity and data protection; in the event of a cyber threat, apply scripts (action protocols) to mitigate it. |
| **D1.C4. LEGAL AND ETHICAL NORMS IN THE DIGITAL ENVIRONMENT** | |
| **DESCRIPTOR** | |
| **Adherence to the principle of integrity, legal and ethical norms, and netiquette.** Knowledge of the rules of use, levels of access, and policies regarding the use of medical data in digital environments.  **Knowledge of key legal provisions concerning the protection of patient rights in eHealth.** Ability to critically evaluate and identify suspicious requests for personal data from third parties; to perform comparison and patient identification; and to apply measures to protect patient rights. Understanding that digital services use privacy policies to inform users about how personal data are processed. Adherence to legal provisions on the protection of patient personal data and on data protection within the EHS as a whole.  **Ability to act in accordance with relevant guidelines, protocols, rules,** and measures regarding the use of various media, information, data, and content, in order to comply with legal, ethical, cultural, and security rules, requirements, and expectations when working with personal, public, professional documents and/or confidential information, data, and digital content. Knowledge and understanding of the legal regulation of the EHS; awareness of how copyright and licensing apply to intellectual property, information, and digital content within and outside the EHS. Knowledge of netiquette. | |
| **Knowledge** | - Knows the rules of conduct concerning the use of medical data in digital environments. - Knows the legal and ethical norms and the principle of integrity in the digital environment. - Knows the legal provisions ensuring the confidentiality of information and the protection of patient personal data within the EHS. - Knows the protocols and rules of conduct in the digital media environment in compliance with legal, ethical, and cultural norms and security rules. - Knows the legal regulation of information dissemination in the EHS and other information systems. - Knows the rules of netiquette. |
| **Skills** | - Adheres to the rules of conduct for the correct and lawful use of medical data in digital environments. - Can assess the legal and ethical consequences of violating integrity rules or netiquette in digital environments. - Can critically evaluate and identify suspicious requests for personal data from third parties that violate netiquette and legal norms. - Adheres to procedures for reporting data security breaches. - Can compare and analyze restricted-access data and information regarding patient identification and the protection of patient rights. - Implements measures to protect patient rights in the digital environment and on internet resources in accordance with legislative acts. - Adheres to legal provisions, the principle of integrity, and ethical norms regarding the protection of patient personal data in MIS and the EHS, and tracks changes in legislation. - Uses legislative acts, recommendations, guidelines, and protocols of organizations and institutions to protect personal rights in the media space of online resources. - Adheres to legislative acts, recommendations, guidelines, and protocols of organizations and institutions to protect patient rights and professional and personal rights in the digital environments of MIS and the EHS. |
| **Attitudes** | - Is aware of the legal consequences of violating integrity and legal and ethical norms when working with personal, public, professional documents and/or confidential information, data, and digital content. - Understands responsibility for using guidelines, protocols, and rules when working with various media, information, data, and content from open and closed sources. - Is aware of how copyright and licensing apply to software products, data, information, and other digital content. - Maintains a responsible attitude toward netiquette and toward legal and ethical norms within and outside the EHS. - Is aware of the diverse needs of users, including persons with disabilities, and adheres to the principles of equality, accessibility, and non-discrimination. |
| **PROFICIENCY LEVELS** | |
| **Level A (Basic)** | **At a basic level, with some autonomy following an instruction or template, or under the guidance of others, can:** comply with legal requirements and regulations related to the processing of personal data, including the requirements of the Law on the Protection of Personal Data and other relevant legislation; adhere to the rules and accepted norms of integrity when carrying out professional duties, using professional information materials, and handling the personal data of colleagues, patients, and others; follow the rules of conduct for the correct and lawful use of medical data in digital environments and online; consciously and with situational awareness assess and identify suspicious requests for personal data from third parties that violate netiquette and legal norms; assess the legal and ethical consequences that may arise from violations of integrity rules, netiquette, or the disclosure of personal data in digital environments. |
| **Level B (Sufficient)** | **At a sufficient level, independently, in line with one's own needs and solving clearly defined standard problems, can:** compare and analyze restricted-access data and information regarding patient identification and the protection of patient rights, applying an individualized approach to typical tasks; implement individual measures to limit the dissemination of personal data of patients and staff in the digital environment, social networks, and other online resources in accordance with legislative acts; adhere to legislative provisions, the principle of integrity, and ethical norms regarding the protection of patient personal data in MIS and the EHS. |
| **Level C (Professional)** | **At a professional level, independently, in line with one's own needs and the needs of others, solving non-standard problems, can:** identify and critically evaluate suspicious, hidden, or complex requests for personal data from third parties that violate netiquette and legal norms regarding the dissemination of the requested information; compare and analyze restricted-access data and information that may be disclosed only with the user's consent, and block attempts by third parties at unlawful patient identification and violation of patient rights; analyze the content of requests and communications on internet resources and social networks for compliance with the norms and rules formally defined in RFC 1855; within the scope of one's competencies and position, use legislative acts, recommendations, guidelines, and protocols of organizations and institutions to protect personal rights in the media space; adhere to legislative acts, recommendations, guidelines, and protocols of organizations and institutions and select the best solutions to protect patient rights and professional and personal rights in the digital environments of MIS and the EHS. |
| **Level D (High)** | **At a high professional or specialized level, including in complex contexts, can:** in cooperation with specialized organizations and experts, develop and propose methodologies and software solutions to identify and counter requests that violate the legal acts governing access to the personal data of patients and healthcare facility staff; recommend refinements to data-handling protocols that may pose threats to the honor, dignity, or rights of users of network resources and the media space; identify and prevent users of shared network resources, shared content, and the digital environment from disregarding the rules of netiquette (RFC 1855). |
| **D1.C5. ASSESSMENT AND IMPROVEMENT OF OWN DIGITAL COMPETENCE** | |
| **DESCRIPTOR** | |
| **Assessment of one's own digital competence; identification and elimination of gaps.** Ability to perform self-assessment, identify gaps, and understand which aspects of one's own digital competence need to be improved or updated; ability to build a personal program for the development of digital competence. Ability to support others in developing their digital competence and to keep pace with the evolution of digital technologies.  **Improvement of one's own digital competence.** Ability to use open digital educational resources (training, courses, educational programs, platforms) for the development of digital competence anywhere and anytime throughout life. Ability to seek opportunities for self-development and continued learning. Ability to manage one's own learning and the development of one's own digital competence using digital technologies and tools. | |
| **Knowledge** | - Knows the Digital Competence Framework for Healthcare Professionals, including its structure, domains, components, and descriptors. - Knows the four proficiency levels of digital competence. - Knows tools for assessing the level of digital competence. - Knows what self-assessment of digital competence is. - Knows which aspects of one's own digital competence need to be improved or updated; can identify gaps and build a personal development program. - Knows how to keep pace with the evolution of digital technologies. |
| **Skills** | - Can perform self-assessment, identify gaps, and understand which aspects of one's own digital competence need to be improved or updated. - Can build a personal program for the development of digital competence. - Can support others in developing their digital competence and keep pace with the evolution of digital technologies. - Can use open digital educational resources (training, courses, educational programs, platforms) to improve digital competence anywhere and anytime throughout life; can seek opportunities for self-development and continued learning. - Can manage and oversee learning and development for oneself and/or others within the healthcare system using digital technologies and tools. |
| **Attitudes** | - Demonstrates and maintains an open attitude toward identifying relevant and innovative digital resources to improve one's own digital competence and the learning of oneself and others. - Is ready to continuously improve one's own digital competence and learning, and to engage new resources and tools that help improve learning effectiveness and achieve new outcomes. |
| **PROFICIENCY LEVELS** | |
| **Level A (Basic)** | **At a basic level, with some autonomy following an instruction or template, or under the guidance of others, can:** communicate with colleagues and the community to identify one's own gaps and assess one's level of digital competence in comparison with others; perform self-assessment or seek a professional assessment from relevant organizations and identify gaps in one's own digital competence; develop or obtain a program for acquiring new qualities, practices, knowledge, and understanding of digital competence; complete a course and/or use available online resources, digital platforms, and environments to improve one's own digital competence. |
| **Level B (Sufficient)** | **At a sufficient level, independently, in line with one's own needs and solving clearly defined standard problems, can:** regularly evaluate one's own digital competence using available assessment tools; build a personal development plan; select appropriate digital educational resources (training, courses, platforms) to address identified gaps; manage one's own learning using digital tools; share information about useful digital educational resources with colleagues. |
| **Level C (Professional)** | **Independently, in line with one's own needs and the needs of others, solving non-standard problems, can:** select and apply differentiated approaches to assessing digital competence depending on the role and tasks of staff; advise colleagues on selecting appropriate digital educational resources and learning pathways; integrate digital learning tools into team workflows; support the development of digital competence among colleagues and contribute to a culture of continuous learning. |
| **Level D (High)** | **At a high professional or specialized level, including in complex contexts, can:** design, propose, and implement institutional programs for the assessment and development of digital competence among healthcare professionals; develop new tools and methodologies for digital competence assessment; lead organizational initiatives that promote digital learning and the systematic improvement of the digital competence of the healthcare workforce. |
| **DOMAIN 2. HEALTH DATA MANAGEMENT** | |
| **D2.C1. WORKING WITH DATA IN THE EHS** | |
| **DESCRIPTOR** | |
| **Management of data, information, and digital content in the Medical Information System (MIS) and the Electronic Health System (EHS).** Understanding of the components of the EHS, its structural organization, and the form in which information is represented within that structure; understanding of the functions of the various elements and components of the EHS; compliance with the requirements for maintaining primary record-keeping forms approved by the Ministry of Health, as well as with the requirements set out in the Procedure for the functioning of the EHS and the procedures for maintaining the relevant registries of the Central Database (CDB) of the EHS, approved by the Ministry of Health.  **Management of medical information and use of other functional capabilities of the EHS;** processing of personal data and other patient information in compliance with the requirements of the Law of Ukraine "On the Protection of Personal Data." Understanding of the interaction between the EHS and other systems and state registries and of the procedure for information exchange between them.  **Data processing.** Ability to determine the type and format of information/data and to make appropriate decisions on how to record and process them in the EHS.  **User skills in the MIS.** As needed, obtaining user access through the MIS operator and registering in the MIS; knowledge of the technical requirements for MIS.  **Ability to operate various patient data elements** for representation in the working (personal) electronic account (electronic referrals; medical certificates of temporary incapacity for work; electronic prescriptions, including those under the "Affordable Medicines" reimbursement program; episodes of medical care and/or diagnostic reports; and other electronic medical records).  **Knowledge and adherence to navigation rules in the MIS,** and to methods of data entry and processing. Ensuring the quality of medical information. Verifying and preparing data for the CDB of the EHS; preparing electronic reports and discharge summaries on the stages, prescriptions, and treatment status of the patient, while complying with ethical requirements and ensuring appropriate data protection and confidentiality. Avoidance of duplication, errors, and inaccuracies in data.  **Reviewing, searching, and filtering patient data.** Ability to formulate information needs and to select, format, store, and organize data and information in the MIS. Performing searches, providing access, organizing, and moving data/information in the MIS. Ability to generate and use a qualified electronic signature (QES) to confirm and protect access to data. | |
| **Knowledge** | - Knows the difference between information and data. - Knows electronic Medical Information Systems (MIS) and the EHS. - Understands the components of the EHS, its structural organization, and the form in which information is represented within that structure. - Knows the functions of the various elements and components of the EHS. - Knows the relationships and the procedure for information exchange between the EHS and state digital registries (such as the Pension Fund of Ukraine and the Civil Registry Office). - Is aware of the requirements for maintaining primary record-keeping forms approved by the Ministry of Health, as well as the requirements set out in the Procedure for the functioning of the EHS and the procedures for maintaining the relevant registries of the CDB of the EHS, approved by the Ministry of Health. - Knows the foundations of the Law of Ukraine "On the Protection of Personal Data." - Knows the regulatory documents governing medical confidentiality and sensitive medical data. - Knows the various types and formats of data and the methods for recording and processing them in the EHS. - Knows the basic technical requirements for MIS, navigation rules in the MIS, and the criteria for the quality of medical information. - Knows the qualified electronic signature (QES) and access protection. |
| **Skills** | - Can manage data, information, and digital content in the MIS and the EHS. - Adheres to the requirements for maintaining primary record-keeping forms in electronic form, approved by the Ministry of Health, as well as the requirements set out in the Procedure for the functioning of the EHS and the procedures for maintaining the relevant registries of the CDB of the EHS. - Can manage medical information and use other functional capabilities of the EHS. - Performs electronic processing of personal data and other patient information in compliance with the requirements of the Law of Ukraine "On the Protection of Personal Data." - Can determine the type and format of information/data and make appropriate decisions on how to record and process them in the EHS. - Possesses user skills in the MIS; can obtain user access and register in the MIS; can create and configure a working/personal workspace in the MIS and register/edit information about oneself in the EHS. - Can operate various patient data elements (electronic referrals; medical certificates of temporary incapacity for work; electronic prescriptions, including those under the "Affordable Medicines" reimbursement program; episodes of medical care and/or diagnostic reports; and other electronic medical records). - Knows how to manage data entry and processing methods and how to ensure the quality of medical information. - Can verify and prepare data for the CDB of the EHS and prepare electronic and, if necessary, paper reports and discharge summaries on the stages, prescriptions, and treatment status of the patient, while complying with ethical requirements and ensuring appropriate data protection and confidentiality. - Can review, search, and filter patient data. - Avoids duplication, errors, and inaccuracies in data. - Can formulate information needs and select, format, store, and organize data and information in the MIS. - Can perform searches, ensure access, organize, and move data/information in the MIS. - Can generate and use a QES to confirm and protect access to data. |
| **Attitudes** | - Acts responsibly to ensure the protection and confidentiality of information. - Pays attention to detail and avoids inaccuracies and duplication of data. - Is flexible toward changes in the MIS and adapts to version updates and the introduction of new functionality. - Is able to quickly adapt skills and knowledge to the various MIS that may be used in different healthcare facilities or healthcare systems. |
| **PROFICIENCY LEVELS** | |
| **Level A (Basic)** | **At a basic level, with some autonomy following an instruction or template, or under the guidance of others, can:** perform basic MIS user functions: as needed, create and configure a personal account/start page in the EHS in line with job competencies and duties; determine the type and format of information/data and make appropriate decisions on how to record and process them in the EHS; process personal data and other patient information in compliance with the Law of Ukraine "On the Protection of Personal Data," review, search and filter, enter, and edit patient data; comply with the requirements for maintaining primary record-keeping forms approved by the Ministry of Health and other healthcare-sector authorities; generate and use a personal QES and ensure its protection from loss. |
| **Level B (Sufficient)** | **At a sufficient level, independently, in line with one's own needs and solving clearly defined standard problems, can:** optimize the configuration of the workplace/personal account in the EHS, including the configuration of privacy and access settings and the protection of access from unauthorized persons; competently manage primary data and the results of patient examinations and diagnostic investigations using the standard built-in menus and services of the user's electronic account; avoid duplication, errors, and inaccuracies; operate various patient data elements (electronic referrals; medical certificates of temporary incapacity for work; electronic prescriptions, including those under the "Affordable Medicines" reimbursement program; episodes of medical care and/or diagnostic reports; and other electronic medical records); verify and prepare data and processed information for transmission to the CDB of the EHS and prepare electronic and, if necessary, paper reports and discharge summaries; formulate search queries and perform addressed and semantic data searches and competently provide rapid access, organization, and movement of data/information in the MIS. |
| **Level C (Professional)** | **At a professional level, independently, in line with one's own needs and the needs of others, solving non-standard problems, can:** ensure the quality of digital processing of primary and diagnostic medical information, results of clinical examinations, prescriptions, and similar; formulate information needs based on patient inquiries and requests from specialist colleagues; select, format, store, and organize data and information in the MIS; select services and resources for generating a QES and use it to confirm and protect access to data. |
| **Level D (High)** | **At a high professional or specialized level, including in complex contexts, can:** develop instructions and other guidance documents on working with the MIS, taking into account its features and other needs; design examination plans that account for diagnostic needs and optimization of the volume of digital data; select, format, store, and organize data and information in the MIS in line with institutional policies and requirements regarding the appropriate volume, form, and duration of storage; analyze the functionality of the MIS and provide recommendations to MIS developers for improving its structural organization and functions. |
| **D2.C2. WORKING WITH DATABASES, STRUCTURES, AND REGISTRIES** | |
| **DESCRIPTOR** | |
| **Awareness of data formats and organization at different levels of the EHS,** and knowledge of the organizational and management model of the functioning of eHealth and of the EHS.  **Knowledge of the features of the MIS interface** used by the healthcare facility, and of the specifics of access to databases, registries, and catalogues.  **Ability to create queries,** generate samples by various parameters, and obtain data from both databases and various registries; process and edit them; enter medical and personal data into the relevant databases and registries; export and import data, as needed, depending on the security settings of each individual database or registry; combine data from different databases and registries into a single document; generate data packages in the form of standardized documents for transfer to colleagues and/or patients; generate discharge summaries for patients from all available data within one's competence; convert data formats for transmission from the MIS to the CDB and back; and use the functionality for issuing electronic referrals and electronic sick leave certificates.  **Awareness of structured and unstructured data,** understanding of the specifics of automating their processing, and awareness of the existence of various types of medical data, in particular health data, and of the methods of obtaining and transmitting them within the MIS, between its components, and across the various levels of the MIS/EHS hierarchy.  **Application of structured data in the planning and modeling of clinical services** and medical practices for improving patient care. Knowledge of the policies governing their use.  **Knowledge and understanding of professional standards** that support the unification of the structure and content of the transmission of medical information. Awareness of the possibility of applying relevant documentation standards when using clinical data.  **Adherence to the policy of using medical data,** with MIS user skills in creating, managing, and sorting databases and preparing various types of reports and recommendations regarding patients, resources/services, or staff workload accounting for a defined period. | |
| **Knowledge** | - Is aware of the format and organization of data at different levels of the EHS; knows the organizational and management model of the functioning of eHealth and of the EHS. - Knows the features of the MIS interface used by the healthcare facility and the specifics of access to databases, registries, and catalogues. - Distinguishes between structured and unstructured data; understands the specifics of automating their processing; is aware of the existence of various types of medical data, in particular health data, and of the methods of obtaining and transmitting them within the MIS. - Knows the policies governing the use of medical data. - Knows and understands professional standards that support the unification of the structure and content of the transmission of medical information. - Is aware of the possibility of applying relevant documentation standards when using clinical data. - Adheres to the policy of using medical data and possesses MIS user skills in creating, managing, and sorting databases and preparing various types of reports and recommendations. |
| **Skills** | - Can create queries and generate samples by various parameters, and obtain data from both databases and various registries. - Can enter medical and personal data into the relevant databases and registries; process and edit them. - Can export and import data, as needed, depending on the security settings of each individual database or registry. - Can combine data from different databases and registries into a single document, ensure data format compatibility, avoid duplication of information, and ensure the clarity and logical structure of the generated document. - Can generate data packages in the form of standardized documents for transfer to colleagues and/or patients. - Can generate registry extracts for patients from all available data within one's competence. - Can convert data for entry into the MIS as needed. - Can use the functionality for issuing electronic referrals, electronic prescriptions, and electronic sick leave certificates. - Applies structured data in the planning and modeling of clinical services and medical practices for improving patient care. - Knows the policies governing their use. |
| **Attitudes** | - Adheres to the policy of using medical data. - Maintains MIS user skills in creating, managing, and sorting databases and preparing various types of reports and recommendations regarding patients, resources/services, or staff workload accounting for a defined period. |
| **PROFICIENCY LEVELS** | |
| **Level A (Basic)** | **At a basic level, with some autonomy following an instruction or template, or under the guidance of others, can:** enter medical and personal data into the relevant databases and registries; process and edit them in line with the needs and tasks within one's job description and professional competencies; create simple standard queries, generate samples by various parameters, and obtain data from both databases and various registries; perform simple data export and import tasks, as needed, depending on the security settings of each individual database or registry; use the functionality for issuing electronic referrals, prescriptions, and sick leave certificates and other electronic documents. |
| **Level B (Sufficient)** | **At a sufficient level, independently, in line with one's own needs and solving clearly defined standard problems, can:** carry out typical tasks of combined access to data from various databases and registries; create and format a single (shared) reporting document on the processing of the generated document; generate data packages in the form of standardized documents for transfer to colleagues and/or patients for further analysis using typical digital technologies; generate extracts from one or more registries for patients from all available data within one's competence and prepare them in accordance with current legal requirements; prevent inconsistencies regarding the form, format, and content of digital documents and current rules, protocols, regulatory acts, and documents; competently use spreadsheets to work with data; create and format data tables for further use in statistical processing packages, in working with EHS databases, and in transferring data to the MIS and CDB. |
| **Level C (Professional)** | **At a professional level, independently, in line with one's own needs and the needs of others, solving non-standard problems, can:** transform/format/organize/structure data in line with protocol requirements for transmission from the MIS to the CDB and back; apply structured data in the planning and modeling of clinical services and medical practices for improving patient care; configure user rights to databases and the parameters of system security or data read/write access within the scope of professional competencies or job description of the healthcare worker. |
| **Level D (High)** | **At a high professional or specialized level, including in complex contexts, can:** design databases and knowledge bases for the needs of a department/clinic/healthcare facility using information technologies for working with relational and non-relational databases of various types; use database management system (DBMS) technology on various operating platforms, primarily of the open-source type; as needed, use the SQL query language to design and configure local databases used within a department/clinic/healthcare facility; effectively work with object-oriented registries and databases in the MIS and EHS using autonomous database technology; optimize resources for storing databases by reducing redundancy and duplication; develop and improve algorithms for efficient management of various data entry and processing methods, using modern digital technologies for signal and image processing. |
| **D2.C3. DATA ANALYSIS IN THE DIGITAL ENVIRONMENT** | |
| **DESCRIPTOR** | |
| **Understanding of the concept of data analysis in the digital environment.** Ability to critically evaluate data and to verify the credibility of information sources and digital content. Ability to verify the correctness, informativeness, relevance, pertinence, and validity of data. Ability to structure and analyze medical data, electronic medical records, electronic referrals, medical reports in the EHS, electronic prescriptions, and similar. Ability to analyze and interpret medical information and data using digital technologies for data sampling and comparison; analysis of data with respect to the concepts of co-reference and interoperability.  **Ability to select and apply digital technologies for data processing and analysis:** typical graphic editors for image processing and specialized editors for the representation, processing, and analysis of data from medical devices and systems.  **Knowledge of the foundations of statistical processing and visualization of medical data** using tables, graphs, and charts in word and graphic processors, for more effective comparative analysis and forecasting of clinical disease trends and the development of epidemics and pandemics. Knowledge and understanding of the relevant data metrics for measuring the effectiveness of healthcare system indicators.  **Mastery of digital data processing technology** using specialized software solutions and information packages. Application, as needed, of methods of correlation, regression, and factor analysis; foundations of taxonomy; and the WHO-recommended triangulation analysis. | |
| **Knowledge** | - Knows what telemedical visualization is, and the technology of obtaining and processing images of the human body, organs, and tissues for clinical analysis, diagnosis, and treatment, including teleradiology, magnetic resonance imaging, ultrasound, endoscopy, elastography, tactile imaging, thermography, medical photography, and similar. - Knows various tools for data analysis, processing, and visualization. - Knows and understands the relevant data metrics for measuring the effectiveness of healthcare system indicators. - Knows the foundations of statistical information processing. - Knows the foundations of healthcare-system indicator analysis and new quantitative strategies, in particular: industrial indicators of the quality of medical care delivery — Pareto efficiency criteria, Taguchi loss criteria, repeatability — Weibull criteria, and others. |
| **Skills** | - Can analyze, critically evaluate, interpret, and verify the correctness and credibility of information sources and digital content. - Can verify the correctness/credibility, informativeness, relevance, pertinence, and validity of data. - Can review, structure, and analyze medical data, electronic medical records, electronic referrals, medical reports in the EHS, electronic prescriptions, and similar. - Can critically evaluate, analyze, and interpret medical information and data using digital technologies for data sampling and comparison. - Can analyze data with respect to the concepts of co-reference and interoperability. - Can select and apply digital technologies for data processing and analysis: typical graphic editors for image processing and specialized editors for the representation, processing, and analysis of data from medical devices and systems. - Has command of digital data processing technology using specialized software solutions and information packages. - Applies, as needed, methods of correlation, regression, and factor analysis; foundations of taxonomy; and the WHO-recommended triangulation analysis. - Can use digital technologies to optimize medical and financial resources and human capacity to meet patient and healthcare-system needs across various levels, including the use of organizational information system software packages. - Can visualize medical statistical data using tables, graphs, and charts in word and graphic processors for more effective comparative analysis and forecasting of clinical disease trends and the development of epidemics and pandemics. |
| **Attitudes** | - Recognizes the importance of structuring and analyzing data for decision-making. - Considers the factor of "transparency" in the use and presentation of data to ensure their reliability, and identifies data presented with particular motives (for example, unethical motives, profit-seeking, manipulation, or misleading). - Pays attention to and monitors data accuracy in evaluations, especially data presented in sophisticated complex forms (for example, attractive complex tables or other visualizations), since the form of data presentation can be used to mislead judgment by creating a false sense of objectivity. |
| **PROFICIENCY LEVELS** | |
| **Level A (Basic)** | **At a basic level, with some autonomy following an instruction or template, or under the guidance of others, can:** systematize and structure primary data; verify the credibility of information sources and digital content received from the patient, from laboratory and clinical observations, or from other resources; analyze digital medical data, electronic medical records, electronic referrals, medical reports in the EHS, electronic prescriptions, and similar; select and apply typical, widespread digital technologies for processing and analyzing input data: typical graphic editors for image processing, XLS spreadsheet processors, and specialized editors for the representation, processing, and analysis of data from devices and systems used in medical practice. |
| **Level B (Sufficient)** | **At a sufficient level, independently, in line with one's own needs and solving clearly defined standard problems, can:** critically evaluate received data and verify the correctness, informativeness, relevance, pertinence, and validity of digital data and information, taking into account their adequacy to the actual clinical case or situation; analyze and interpret digital medical information and data using digital technologies for data sampling and comparison; analyze data for compatibility across the set of obtained results, with respect to the concepts of co-reference of medical records and the interoperability of data based on the technical solutions used to obtain, store, and transmit them; apply in-depth data analysis through additional information using the capabilities of standard licensed or open-access software packages. |
| **Level C (Professional)** | **At a professional level, independently, in line with one's own needs and the needs of others, solving non-standard problems, can:** perform comparative analysis of multiple data sources; design integrated approaches to data analysis using statistical software (for example, SPSS, R, Stata) and specialized medical analytical packages; apply correlation, regression, and factor analysis methods to support clinical and managerial decisions; develop visualization solutions tailored to specific audiences (clinicians, managers, patients); evaluate the quality of data and the validity of analytical conclusions; mentor colleagues in the use of analytical tools. |
| **Level D (High)** | **At a high professional or specialized level, including in complex contexts, can:** design and lead complex data analysis projects in healthcare; develop methodologies for evaluating healthcare system performance using advanced quantitative strategies; integrate data from heterogeneous sources for population-level analysis; oversee analytical workflows in healthcare facilities; contribute to the development of national health analytics policies and standards; propose innovations in the application of data analytics in healthcare. |
| **D2.C4. WORKING WITH CLINICAL CODES AND CLASSIFIERS** | |
| **DESCRIPTOR** | |
| **Knowledge and use of clinical coding terminology,** clinical code databases and classifiers, and clinical protocols of patient conditions; the ability to sort them in the digital environment and to verify data using codes and classifiers. Use of digital medical reference works and disease classifiers, including ICD and ICD-11; LOINC (Logical Observation Identifiers Names and Codes) — a database and universal standard for identifying medical and laboratory observations; the International Classification of Primary Care, Second Edition; and similar resources. Application of PGDs (Patient Group Directions) and PSDs (Patient Specific Directions); use of clinical protocols oriented toward various medical conditions, such as e-TB Manager, HIV/AIDS solutions, and prescription guidance for various patient groups. Ability to organize and structure medical/biomedical data. Digital skills in pharmacy and medication management for prescriptions issued through electronic prescriptions: prescribing medications electronically; using electronic prescription and medication management systems to minimize errors and improve patient safety; collecting, analyzing, and interpreting large datasets for informed decision-making in pharmaceutical supply chain management; integrating medication search systems, storage, and online ordering with existing processes; familiarity with how the pharmaceutical logistics system, the state procurement system, and the distribution of medications across healthcare facilities, write-off, and reimbursement processes operate. Understanding of the principles of organizing and structuring medical/biomedical information through ontologies: SNOMED, UMLS (Unified Medical Language System), FMA (Foundational Model of Anatomy), and other ontologies. | |
| **Knowledge** | - Knows who owns the registry data. - Knows the features of the user interface for entering and storing coded information and data. - Knows the terminology of clinical coding, clinical code databases and classifiers, and clinical protocols of patient conditions. - Knows and understands the requirements for collecting digital data to support clinical coding and data management processes. - Uses coding in the MIS to record key patient information (for example, diagnoses, allergies, procedures, adverse drug reactions). - Knows electronic prescription systems and medication management systems. - Knows medication search, storage, and online ordering systems. - Understands the principles of organizing and structuring medical/biomedical information; knows the ontologies SNOMED, UMLS (Unified Medical Language System), FMA (Foundational Model of Anatomy), and other ontologies. - Recognizes that the foundation of contemporary knowledge for healthcare professionals includes the following ontologies: SNOMED, UMLS, FMA, and others. |
| **Skills** | - Can practically use clinical coding terminology, clinical code databases and classifiers, and clinical protocols of patient conditions; sort them in the digital environment; and verify data using codes and classifiers. - Can use digital medical reference works, disease classifiers including ICD and ICD-11, as well as LOINC (Logical Observation Identifiers Names and Codes), the International Classification of Primary Care (Second Edition), and similar resources. - Applies PGDs/PSDs and clinical protocols oriented toward various medical conditions — e-TB Manager registries, HIV/AIDS solutions, prescription guidance for various patient groups, and similar. - Can organize and structure medical/biomedical data. - Has digital skills in medical practice related to pharmacy and electronically prescribed medications: can prescribe medications via electronic prescriptions; knows how to use electronic prescription systems and medication management systems to minimize errors and improve patient safety. - Can collect, analyze, and interpret large datasets for informed decision-making in pharmaceutical supply chain management. - Can integrate medication search systems, storage, and online ordering with existing processes. - Is familiar with how the pharmaceutical logistics system, the state procurement system, the distribution of medications across healthcare facilities, and write-off and reimbursement processes operate. |
| **Attitudes** | - Confidently and consciously uses clinical coding terminology and clinical code databases and classifiers. - Pays attention to detail. - Considers the protection of patient data and adheres to the relevant laws and rules concerning data confidentiality and security. |
| **PROFICIENCY LEVELS** | |
| **Level A (Basic)** | **At a basic level, with some autonomy following an instruction or template, or under the guidance of others, can:** practically use clinical coding terminology and clinical code databases and classifiers in healthcare within the scope of one's competence and job description for working with patients at the primary care level; maintain clinical protocols of patient conditions, sort and search information; track completeness and timely entry of data according to the nomenclature of registries, codes, and classifiers related to medical activities, in the relevant digital environment used by the clinic/facility; implement basic digital skills in the digital processing of information related to coding and classification of data in pharmacy, medication management, electronic prescriptions, and the logistical support of pharmaceutical institutions; monitor the updating and currency of information and registries of medical and technological documents according to one's professional area. |
| **Level B (Sufficient)** | **At a sufficient level, independently, in line with one's own needs and solving clearly defined standard problems, can:** use digital medical reference works and disease classifiers, including ICD and others, when conducting clinical examinations, diagnostics, and patient monitoring with the available tools of the EHS; as needed, use available international standardized systems of codes, classifiers, and registries (for example, LOINC, the International Classification of Primary Care, Second Edition); conduct advanced searches and update information on registries of treatment protocols according to one's professional area; use the electronic system for recording prescription drug orders and monitoring drug availability and possible substitutions; use electronic prescription and medication management systems to minimize errors and improve patient safety. |
| **Level C (Professional)** | **At a professional level, independently, in line with one's own needs and the needs of others, solving non-standard problems, can:** implement digital skills in medical practice related to the provision of primary or emergency care, diagnostics, pharmacy, and electronically prescribed medications using relevant up-to-date classifiers; conduct in-depth searches in digital sources and correctly use information from classifiers of laboratory tests and indicators and sectoral classifiers of medical procedures in professional practice; track the currency and updates of codes, registries, and classifiers of medical care in international information resources. |
| **Level D (High)** | **At a high professional or specialized level, including in complex contexts, can:** fluently operate information available in the digital environment regarding clinical PGDs/PSDs protocols oriented toward various medical conditions — e-TB Manager registries, HIV/AIDS solutions, and similar; registries of medical and technological documents on the standardization of medical care due to the need to localize the spread of epidemic conditions, pandemics, and similar (for example, those caused by COVID-19); propose solutions for updating or improving the recording of guidelines and registries of protocols for prescriptions for various patient groups based on analysis of digital information on the recording and registration of features of various clinical cases; collect, analyze, and interpret large datasets for informed decision-making on pharmaceutical supply chain management, improving logistics, accounting, and use; competently integrate medication search, storage, and online ordering systems with existing processes in the clinic/institution/healthcare facility; using professional experience and a thorough command of up-to-date information on the functioning of the pharmaceutical logistics system, the state procurement system, and the distribution of medications across healthcare facilities, write-off, and reimbursement processes, propose ways to optimize resource allocation and improve healthcare facility activities, including through software packages of organizational information systems. |
| **D2.C5. WORKING WITH OTHER COMPONENTS OF UKRAINE'S eHEALTH** | |
| **DESCRIPTOR** | |
| Knowledge of the structures and organizational and management models of the information ecosystem of Ukraine's healthcare (eHealth and the Electronic Health System); awareness of the functional purpose, features, and goals of the use of various information systems; ability to classify components of the ecosystem (local, regional, specialized, and similar), determine their subjectivity/affiliation/subordination and the tasks they address; ability to format and organize data in line with the requirements of various MIS; knowledge of the rules of using various information systems (registration, data entry, creating data requests, and similar); avoidance of errors in medical queries; critical evaluation of the appropriateness of submitting information to a particular information system; submission of statistical information and data to relevant authorities and systems; understanding of the requirements and specifics of ensuring data interoperability as the technological compatibility of information technology solutions for the cross-accessibility of digital records in various information systems used in Ukraine's eHealth and/or abroad. | |
| **Knowledge** | - Knows the structure and the organizational and management model of the information ecosystem of Ukraine's healthcare (eHealth and the EHS). - Is aware of the functional purpose, features, and goals of the use of various information systems. - Critically evaluates the appropriateness of submitting information to a particular information system. - Submits statistical information and data to relevant authorities and systems. - Performs intersectoral coordination with various components of eHealth using digital technologies. - Understands the requirements and specifics of ensuring data interoperability as the technological compatibility of information technology solutions for the cross-accessibility of digital records in various information systems used in Ukraine's eHealth and/or abroad. |
| **Skills** | - Can classify components of the ecosystem (local, regional, specialized, and similar), determine their subjectivity/affiliation/subordination and the tasks they address. - As needed, can format and organize data in line with the requirements of the CDB and the relevant MIS interface used in the healthcare facility. - Knows how to use various information systems (registering, entering data, creating data requests, and similar). - Avoids errors in medical queries. - Submits statistical information and data to relevant authorities and systems. - Can perform intersectoral coordination with other components of eHealth using digital technologies. |
| **Attitudes** | - Acts responsibly when submitting statistical information and data to relevant authorities and systems. - Critically evaluates the appropriateness of submitting information to a particular information system. - Strives to make effective use of the capabilities of eHealth. - Is attentive to online service providers, verifies the reliability of these providers, and avoids exposure to online dangers when receiving online services. |
| **PROFICIENCY LEVELS** | |
| **Level A (Basic)** | **At a basic level, with some autonomy following an instruction or template, or under the guidance of others, can:** classify components of the eHealth ecosystem (local, regional, specialized, and similar), determine their subjectivity/affiliation/subordination and the tasks they address; identify the data and information they handle and their interconnections with other eHealth components; use specialized MIS within the scope of the job description; avoid errors and inaccuracies in medical queries. |
| **Level B (Sufficient)** | **At a sufficient level, independently, in line with one's own needs and solving clearly defined standard problems, can:** process queries received by specialized MIS from the EHS, the National Health Service of Ukraine (NHSU), and other organizations; submit specialized statistical information and data to the relevant authorities and systems; professionally use medical reference, consultative-diagnostic, and other specialized medical systems (for example, Tsentral-103, e-Krov, and similar) at the basic and specialized levels; work with specialized hardware-software complexes and technical systems for specialized purposes; process laboratory, diagnostic, monitoring, and other information in line with the professional area of the healthcare worker; activate and protect the automated workplace of the physician from unauthorized access by third parties; connect auxiliary devices and activate the necessary services of the specialized MIS. |
| **Level C (Professional)** | **At a professional level, independently, in line with one's own needs and the needs of others, solving non-standard problems, can:** perform intersectoral coordination with other domains and components of eHealth using digital technologies; effectively use information banks of medical institutions and services, local MIS of treatment-and-prevention institutions, and screening information systems for primary preventive examinations of the population; systematize the data obtained in such systems for further processing and modeling using modern information technologies and specialized software resources; competently adapt and use the functionality of specialized workstations of the software complexes "Stationary," "Polyclinic," "Emergency Care," "Drug Accounting," "Accounting Calculations," "Personnel," and other hospital information system packages to address local healthcare facility tasks. |
| **Level D (High)** | **At a high professional or specialized level, including in complex contexts, can:** use territorial-level MIS to manage specialized medical services, collect and systematize data at the territorial level, work with the functionality of administrative and management MIS, and implement automated technologies for managing healthcare facilities; apply specialized software packages for in-depth analysis and statistical processing of data at the territorial level; use specialized MIS of research institutions and medical educational institutions for processing databases, automating R&D activities, management functions, organizational matters, and the automation of the recording of teaching and student activities at higher education institutions. |
| **DOMAIN 3. DIGITAL COMMUNICATION AND TRANSFER OF CARE** | |
| **D3.C1. PROFESSIONAL DIGITAL COMMUNICATION** | |
| **DESCRIPTOR** | |
| **Sharing and exchanging data using digital technologies** at the local level — in the laboratory/department/section/healthcare facility/institution. Use of appropriate digital tools and technologies for professional communication and for sharing and exchanging data, information, and digital medical content. Knowledge of practical methods of citing primary sources, adherence to the rules of confidentiality and copyright protection, and the ability to act responsibly. Ability to communicate effectively with patients, other healthcare professionals, and other stakeholders involved in patient care via digital communication. Ability to send electronic messages; use communication technologies such as email, messengers, video conferencing, and other digital tools to communicate with patients and other healthcare providers. Use of various means of communication to organize digital communication: synchronous communication (in real time) and asynchronous communication (non-simultaneous communication, such as via email or a forum for sending messages), in one-to-one, one-to-many, or many-to-many modes. Effective communication with patients online using audience-specific communication strategies and various means, taking into account language, age, cultural, and social differences, features, and limitations, while respecting their autonomy and supporting informed decision-making. Knowledge of the main risks and uncertainties in professional digital communication in healthcare; ability to use modern online sources of scientific medical information for professional activities. | |
| **Knowledge** | - Knows digital tools and technologies for professional electronic communication and for sharing and exchanging data, information, and digital medical content. - Knows communication technologies such as email, messengers, video conferencing, and other digital tools for communication. - Knows synchronous communication (in real time) and asynchronous communication (non-simultaneous communication, such as via email or a forum for sending messages), in one-to-one, one-to-many, or many-to-many modes. - Knows the target audiences of professional communication and takes into account language, age, cultural, and social differences, features, and limitations, while respecting their autonomy and supporting informed decision-making. - Knows the basic concepts of risks and uncertainties in professional digital communication in healthcare. - Knows modern online sources of scientific medical information for use in professional activities. |
| **Skills** | - Can exchange data using digital technologies at the institutional level — in the laboratory/department/section/healthcare facility/institution. - Uses appropriate digital tools and technologies for professional communication and for sharing and exchanging data, information, and digital medical content. - Acts as an intermediary, knows practical methods of citing primary sources, adheres to the rules of confidentiality and copyright protection, and acts responsibly. - Can communicate effectively with patients, other healthcare professionals, and other stakeholders involved in patient care via digital communication. - Uses communication technologies such as email, messengers, video conferencing, and other digital tools to communicate with patients and other healthcare providers. - Can use various means of communication to organize digital communication: synchronous (real time) and asynchronous (non-simultaneous, for example via email or a forum), in one-to-one, one-to-many, or many-to-many modes. - Can communicate effectively with patients online using audience-specific strategies and various means, taking into account language, age, cultural, and social differences, features, and limitations, while respecting their autonomy and supporting informed decision-making. - Can use modern online sources of scientific medical information for professional activities. |
| **Attitudes** | - Is willing to listen to others and confidently engage in online conversations, achieving clarity and mutual understanding in both personal and social contexts. - Is open to artificial intelligence and machine learning systems that help people make informed decisions in line with their goals (for example, users actively decide whether or not to act on a physician's recommendation). - Is willing to adapt the communication strategy to the situation and digital tools: verbal strategies (written, oral language), non-verbal strategies (body language, facial expressions, tone of voice), visual strategies (signs, icons, illustrations), or mixed strategies. - Adheres to the rules of confidentiality and copyright protection and acts responsibly. |
| **PROFICIENCY LEVELS** | |
| **Level A (Basic)** | **At a basic level, with some autonomy following an instruction or template, or under the guidance of others, can:** use various means of communication to organize professional digital communication, in particular, send electronic messages; use email, messengers, and video conferencing systems; use online sources of scientific medical information for professional activities. |
| **Level B (Sufficient)** | **At a sufficient level, independently, in line with one's own needs and solving clearly defined standard problems, can:** use appropriate digital tools and communication technologies for professional communication with various target groups; possess the technique of correct online communication and reasoned discussion; find and use online sources of scientific medical information for professional activities. |
| **Level C (Professional)** | **At a professional level, independently, in line with one's own needs and the needs of others, solving non-standard problems, can:** use appropriate digital tools and technologies for professional communication and for sharing and exchanging data, information, and digital medical content; use digital technologies to improve organizational communication with process participants; create discussion topics and groups (open and closed) to discuss current issues; join existing online thematic groups; manage groups in social networks or messengers; communicate effectively with patients, other healthcare professionals, and other stakeholders involved in patient care via digital communication; adhere to the rules of using online resources and copyright protection. |
| **Level D (High)** | **At a high professional or specialized level, including in complex contexts, can:** communicate effectively with patients and colleagues online, using various audience-specific communication strategies and various means, taking into account language, age, cultural, and social differences, features, and limitations; contribute to the joint development and improvement of organizational communication strategies; adapt communication strategies to the situation and digital tools for a specific target audience. |
| **D3.C2. DIGITAL INTERACTION** | |
| **DESCRIPTOR** | |
| **Ability to use digital tools for remote interaction to achieve common results and goals in professional activities,** to interact online with other healthcare professionals, such as specialists and nurses, to provide coordinated and comprehensive patient care (electronic referrals for additional examinations, consultations, and similar); as needed, to interact with patients' families and caregivers to provide them with information and support. Ability to interact effectively with other stakeholders, such as insurance companies, government institutions, and healthcare organizations; to conduct document workflows electronically (including the exchange of contracts between medical institutions and the NHSU, which must also be signed electronically). Ability to analyze scientific statistical information and adapt it for interaction with non-professional audiences. Knowledge of the basic principles of disseminating information and disinformation in modern digital communication tools, and the ability to take this knowledge into account for informing on health issues and countering health-related disinformation. Use of appropriate digital tools and technologies for professional interaction; understanding of which means of electronic communication are appropriate for the given context. Ability to demonstrate and uphold an ethical, positive, sensitive, and responsible attitude and behavior during online interaction. | |
| **Knowledge** | - Knows digital tools and technologies for sharing and exchanging data, information, and digital content; digital tools for remote interaction (both at the local/institutional level — in the laboratory/department/section/healthcare facility/institution — and through open and closed channels of network communication, including the available channels of information exchange in the MIS and EHS). - Knows strategies and methods of interacting with patients and colleagues using digital tools, including tools for joint work on projects and the exchange of documents and work-related information. - Knows electronic document workflow (including contracts between medical institutions and the NHSU, which must also be signed electronically). - Knows the foundations of scientific statistical information and how to adapt it for non-professional audiences. - Knows the basic principles of disseminating information and disinformation in modern digital communication tools; knows what an infodemic is; knows methods of countering disinformation. - Knows how to use digital solutions for interaction in disaster zones and/or emergency situations. |
| **Skills** | - Can interact with others using digital technologies and tools to achieve common results and goals. - Can use digital tools for remote interaction (both at the local/institutional level and through open and closed channels of network communication, including the available channels of information exchange in the MIS and EHS). - Can interact online with other healthcare professionals, such as specialists and nurses, to provide coordinated and comprehensive patient care (electronic referrals for additional examinations, consultations, and similar); as needed, can interact with patients' families and caregivers to provide them with information and support. - Can interact effectively with other stakeholders, such as insurance companies, government institutions, and healthcare organizations. - Can conduct document workflows electronically (including contracts between medical institutions and the NHSU, which must also be signed electronically). - Can analyze scientific statistical information and adapt it for non-professional audiences. - Knows the basic principles of disseminating information and disinformation in modern digital communication tools; can take this knowledge into account for informing on health issues and countering health-related disinformation. - Uses appropriate digital tools and technologies for sharing and exchanging data, information, and digital content. - Interacts using a wide range of digital technologies and understands which means of electronic communication are appropriate for the given context. - Can demonstrate and uphold an ethical, positive, sensitive, and responsible attitude and behavior in online collaboration. - Interacts using a wide range of digital technologies and understands which means of digital communication are appropriate for the given context (in terms of cultural, social, and gender-specific differences and similar). - Implements joint online projects and social initiatives. - As needed, can communicate online with patients' families and caregivers to provide them with information and support. |
| **Attitudes** | - Demonstrates and upholds an ethical, positive, sensitive, and responsible attitude and behavior in online collaboration. - Acts in a reliable manner to achieve group goals when participating in the joint creation of resources or knowledge. - Is inclined to use appropriate digital tools to facilitate interaction among team members while ensuring digital accessibility. |
| **PROFICIENCY LEVELS** | |
| **Level A (Basic)** | **At a basic level, with some autonomy following an instruction or template, or under the guidance of others, can:** interact online with other healthcare professionals to provide coordinated and comprehensive patient care. |
| **Level B (Sufficient)** | **At a sufficient level, independently, in line with one's own needs and solving clearly defined standard problems, can:** interact online with patients, colleagues, and all stakeholders, such as insurance companies, government institutions, and healthcare organizations; conduct document workflows electronically (including contracts between medical institutions and the NHSU, which must also be signed electronically). |
| **Level C (Professional)** | **At a professional level, independently, in line with one's own needs and the needs of others, solving non-standard problems, can:** use appropriate digital tools and technologies for sharing and exchanging data, information, and digital content; interact using a wide range of digital technologies and understand which means of electronic communication are appropriate for the given context; use digital tools for remote interaction (both at the local/institutional level — in the laboratory/department/section/healthcare facility/institution — and through open and closed channels of network communication, including the available channels of information exchange in the MIS and EHS); know how to use digital solutions for interaction in disaster zones and/or emergency situations; participate in joint online projects and initiatives. |
| **Level D (High)** | **At a high professional or specialized level, including in complex contexts, can:** interact online; know the basic principles of disseminating information and disinformation in modern digital communication tools and take this knowledge into account for informing on health issues and countering health-related disinformation; initiate and implement joint online projects and social initiatives. |
| **D3.C3. COLLABORATION IN THE DIGITAL ENVIRONMENT** | |
| **DESCRIPTOR** | |
| **Telemedicine, telemedical consultation, remote diagnostics, and treatment.** Ability to apply digital technologies and tools for collaboration and for achieving faster and more effective delivery of medical services and care through telemedicine at a distance. Ability to collaborate effectively, address joint tasks, and work synchronously or asynchronously on a shared problem in a professional digital environment (physician–physician, physician–patient) using stationary and mobile systems in real time (synchronously) or with delay (asynchronously). Use of telemedical consultation platforms and complexes for: telemedical consultations, telemedical case conferences, telemetry, home teleconsultation, and the performance of medical manipulations and operations. Compliance with Ukrainian legislation on telemedicine. Collaboration in the digital environment may include, but is not limited to, performing remote diagnostics, monitoring complex medical manipulations using real-time video, remote monitoring of patient status during the postoperative period or rehabilitation, monitoring and, as needed, telepresence/teleassistance to achieve common goals, telenursing (the ability to establish patient–nurse relationships based on assessment, planning, and investigation of patient needs), and collaboration with other medical services for high-quality care delivery, and similar. Disease prevention and emergency response in healthcare: communication with the population on healthy lifestyles using digital technologies; raising the medical literacy of citizens; monitoring of behavioral health determinants, rumors, and disinformation at the national, regional, and community levels; support of effective response systems (algorithms, channels, human resources) for emergencies; provision of disaster medicine services (qualified medical care in disaster sites, patient triage, and similar). | |
| **Knowledge** | - Knows telemedicine, the Law on Telemedicine, remote diagnostics, and digital consultation and treatment. - Knows telemedical methods and telemedical consultation platforms. - Knows the organization of online communications and consultations: physician–physician, physician–patient, teleconsultations, and telemedical case conferences. - Knows e-summaries (electronic medical conclusions), e-medical orders, and e-prescriptions. - Knows the principles of remote monitoring of patient status. - Knows home telemedicine and telenursing. - Knows remote operation and telepresence/teleassistance. |
| **Skills** | - Can apply digital technologies and tools for collaboration and for achieving faster and more effective delivery of medical services and the application of telemedical methods at a distance. - Can interact effectively in a professional digital environment using telemedical consultation platforms, organizing online communications and consultations physician–physician and physician–patient via stationary and mobile systems in real time or with delay. - Can conduct teleconsultations and telemedical case conferences, perform remote diagnostics, provide e-reports and expert advice, and issue remote e-prescriptions. - Can effectively use information panels or dashboards for the visualization and analysis of data, allowing healthcare professionals to quickly assess and compare results and trends in medical practice. - Can perform complex medical manipulations (as needed, remote operation, telepresence/teleassistance) to achieve common goals using real-time video and provide other medical services through telemedicine. - Can perform remote monitoring of patient status during the postoperative period or rehabilitation. - Can issue e-discharge summaries and e-prescriptions during virtual consultations and video conferences. - Can provide home telemedicine and telenursing services. - Adheres to the provisions of the Law on Telemedicine. |
| **Attitudes** | - Encourages everyone to express their views constructively during collaboration in the digital environment. - Acts in a reliable manner to achieve group goals during interaction in joint projects, teleconsultations, and similar. - Is willing to convey one's reasoned opinion or decision constructively and concisely. - Is inclined to use appropriate digital tools to facilitate collaboration among team members while ensuring digital accessibility. |
| **PROFICIENCY LEVELS** | |
| **Level A (Basic)** | **At a basic level, with some autonomy following an instruction or template, or under the guidance of others, can:** comply with the basic provisions of the Law on Telemedicine when communicating and collaborating on shared tasks, projects, and similar; use modern personal telecommunication tools and gadgets, hardware-software technical means, to ensure reliable communication and quality connection between healthcare facility staff and the patient/contact person; provide a defined list of services using digital technologies of home telemedicine and telenursing services; correctly and benevolently establish patient–nurse relationships based on a patient-oriented approach grounded in assessment, planning, and investigation of patient needs; conduct teleconsultations and telemedical case conferences; perform remote diagnostics and, on their results, provide and substantiate electronic conclusions, expert recommendations, and advice; perform remote electronic prescriptions. |
| **Level B (Sufficient)** | **At a sufficient level, independently, in line with one's own needs and solving clearly defined standard problems, can:** interact effectively in a professional digital environment using high-quality, high-clarity telemedical consultation platforms capable of ensuring reliable connection and the organization of online physician–physician and physician–patient communications and consultations via stationary and mobile systems in real time or with delay; apply digital technologies and tools to transmit data to specialists for additional clinical investigations and clarification of the diagnosis, conduct treatment activities, and collaborate to achieve faster and more effective delivery of medical services; perform remote monitoring of patient status before or after surgery or during rehabilitation; conduct electronic document workflow, generate e-discharge summaries, and issue e-prescriptions during virtual consultations and video conferences. |
| **Level C (Professional)** | **At a professional level, independently, in line with one's own needs and the needs of others, solving non-standard problems, can:** perform complex medical manipulations (as needed, ensure remote telepresence/teleassistance) to achieve common goals using real-time video and provide other medical services through telemedicine; apply communication and information resources and digital skills for cooperation with various other medical services and healthcare institutions for high-quality care delivery and the resolution of social inquiries; through digital telecommunication means and real-time video, perform long-term monitoring of complex medical manipulations; create shared digital resources within a clinic/institution/facility for further use to improve remote diagnostics and/or care delivery to patients. |
| **Level D (High)** | **At a high professional or specialized level, including in complex contexts, can:** communicate with the population on healthy lifestyles and raise the digital and medical literacy of citizens through trainings and webinars, with the use of digital technologies and telecommunication and computer means; monitor on social networks and in the media the behavioral determinants of health, rumors, and disinformation at the national, regional, and community levels; maintain and implement effective information-and-organizational systems (algorithms, channels, human resources) for response to emergencies, technogenic/anthropogenic impacts, and natural disasters in order to protect and preserve the health of citizens. |
| **D3.C4. DATA EXCHANGE IN THE EHS (declarations, medical episodes, electronic referrals, medical certificates, electronic prescriptions, and similar)** | |
| **DESCRIPTOR** | |
| **Interaction between the MIS and the CDB.** Knowledge and execution of the general rules for generating medical records, creating cases/episodes, medical certificates, electronic prescriptions, and referrals. Ability to transfer clinical information, examination results, and physician referrals; assess risks in data transfer; and formalize and carry out the preparation and exchange of data within the healthcare facility and the healthcare system as a whole. Transfer of personal data and examination results for the provision of care to patients by third parties. Ability to register declarations on the choice of a primary care physician. Ability to edit a patient's registration (passport) data in the EHS. | |
| **Knowledge** | - Knows eHealth, the EHS, the CDB, the MIS, the electronic Medical Information System, the state enterprise "Electronic Health," software modules, registries, the electronic account, electronic identification, verification, electronic cases/episodes, electronic medical records, electronic medical certificates, e-prescriptions, e-referrals, risks in data transfer, protection of information from unauthorized access, destruction, and modification, personal data, and the transfer of examination results for the provision of care to patients by third parties; storage, automatic backup, and recovery of data. - Can register declarations of patients with primary care physicians and edit a patient's registration (passport) data in the EHS. - Knows the Law of Ukraine "On the Protection of Personal Data." - Knows the unified standards of medical information exchange approved by the Ministry of Health. - Knows the procedure for the functioning of the EHS. - Knows who manages the CDB and who is the owner, manager, and administrator of the registry information of the CDB. - Knows how to register in the EHS, obtain access rights, and verify. - Is aware of the main objectives of the EHS — namely, ensuring patients' use of electronic services to exercise their rights, particularly under the program of state guarantees of medical care for the population (the medical guarantees program); the automation of accounting for medical services and the management of medical information; and the introduction of electronic document workflow in the medical care of the population. |
| **Skills** | - As needed, can register in the central database of the EHS and submit applications/requests to enter changes, additions, and clarifications regarding personal information about oneself in the Registry of Medical Specialists. - As needed, can register a patient in the Patient Registry at the request of the patient or the patient's legal representative, and submit applications to enter changes and additions to information about the patient in the Patient Registry. - Can enter into the CDB and, as needed, edit records about patient registration (passport) data. - Can submit to the central database a request to register a record in the Registry of Declarations on the Choice of a Primary Care Physician. - Can create and enter into the central database information about prescriptions, referrals, and other medical records; review and submit applications to enter changes and additions to documents and information entered into the central database. - Can submit requests and obtain access to data about the patient in the central database for the purposes of healthcare, establishing a medical diagnosis/conclusion, ensuring treatment or the provision of medical services, and the functioning of the EHS, while complying with the requirements of the legislation on medical confidentiality and ensuring the protection of personal electronic medical data. - Can search and review information in the central database in line with the user's access rights, while complying with the requirements of the Law of Ukraine "On the Protection of Personal Data." - Can conclude, modify, and terminate contracts on the medical care of the population and contracts on reimbursement under the medical guarantees program; generate and submit electronic reports, primary, settlement, and other documents under the contracts via the central database of the EHS. - Can use national classifiers, nomenclatures, and reference works approved in accordance with the procedure established by law for entering and systematizing information in the central database. - Can use the automated system for tracking operations (entry, review, changes, additions, and similar) with information and documents in the central database and events occurring in the EHS that relate to its security. |
| **Attitudes** | - Adheres to the unified standards of medical information exchange approved by the Ministry of Health. - Is aware of the necessity to protect data that constitute medical confidentiality — the list of such data, the procedure for accessing them, and the cases of transferring them to third parties. - Carefully processes any information, ensuring the protection of data from unauthorized access, destruction, and modification. |
| **PROFICIENCY LEVELS** | |
| **Level A (Basic)** | **At a basic level, with some autonomy following an instruction or template, or under the guidance of others, can:** acquire digital skills for working with resources, databases, and registries in the MIS and the CDB of the EHS; transfer data for registration in the central database of the EHS; as needed, at the request of the patient or the patient's legal representative, register the patient or submit applications to enter changes and additions to information about the patient in the Patient Registry; submit digital requests and obtain access to data about the patient in the central database for the purposes of healthcare, establishing a medical diagnosis/conclusion, ensuring treatment or the provision of medical services, and the functioning of the EHS, in compliance with the legislation on medical confidentiality and ensuring the protection of personal data. |
| **Level B (Sufficient)** | **At a sufficient level, independently, in line with one's own needs and solving clearly defined standard problems, can:** work with the digital resources of the MIS and the CDB to enter requests into the central database to register a record in the Registry of Declarations on the Choice of a Primary Care Physician; create digital content and enter into the central database information about prescriptions, referrals, and other medical records; review and submit applications to enter changes and additions to documents and information entered into the central database by oneself or other users, with the appropriate authorization; conclude, modify, and terminate contracts on the medical care of the population and contracts on reimbursement under the medical guarantees program; generate and submit digital electronic reports, primary, settlement, and other documents under the contracts via the central database of the EHS. |
| **Level C (Professional)** | **At a professional level, independently, in line with one's own needs and the needs of others, solving non-standard problems, can:** process any digital information while ensuring adherence to the basic principles, requirements, and technologies of cyber protection and cyber hygiene implemented in the components of the EHS to protect data from unauthorized access, destruction, and modification; use advanced skills in working with databases and digital content to enter and systematize information in the central database of the EHS; submit applications/requests to enter changes, additions, and clarifications regarding personal information in the Registry of Medical Specialists; effectively and correctly use national classifiers, nomenclatures, and reference works approved in accordance with the procedure established by law for the formation of digital content of individual components and documents of the EHS; adhere to the unified standards approved by the Ministry of Health for the exchange of digital data and medical information in the MIS and the EHS. |
| **Level D (High)** | **At a high professional or specialized level, including in complex contexts, can:** use the automated system for tracking operations (entry, review, changes, additions, and similar) with information and documents in the central database and events occurring in the EHS that relate to its security. |
| **D3.C5. INTERACTION WITHIN UKRAINE'S HEALTHCARE ECOSYSTEM (between the EHS and other electronic systems)** | |
| **DESCRIPTOR** | |
| **Ability to interact between various information systems to address professional issues,** and awareness of the need to escalate problem resolution; interaction with state registries (electronic sick leave certificates, pension funds); ability to provide feedback outside the EHS; ability to use communication modules and technical support systems of various MIS; timely communication regarding cases of infectious diseases and interaction to prevent epidemic outbreaks; online interaction in the provision of emergency medical care (creating medical records/dispatching/forming teams, and similar); ability to interact with the information systems of the National Health Service of Ukraine (NHSU) for processing contracts and agreements with healthcare facilities, with the Information System for Monitoring Socially Significant Diseases, with the Pension Fund of Ukraine, and similar; online interaction with a group of specialized databases and information systems (for example, the electronic system for managing stocks of medicines and medical products "e-Stock," critical registries, and similar), as well as with niche professional systems at the local level; interaction with other organizations in Ukraine and with international information systems. | |
| **Knowledge** | - Is aware of the components of the eHealth ecosystem. - Knows the main communication modules and technical support systems of various MIS. - Knows how electronic medical records are created, how dispatching is carried out, how teams are formed, and similar. - Knows how to quickly interact with Tsentral 103, Centers for Emergency Medical Care (CEMC), and similar. - Knows the information systems of the NHSU for processing contracts and agreements with healthcare facilities and the Information System for Monitoring Socially Significant Diseases. - Knows the electronic system for managing stocks of medicines and medical products "e-Stock," e-Krov, the embryological system, e-CPD, critical registries, and similar. - Is aware of the existence of major international information systems. |
| **Skills** | - Can interact between various information systems to address professional issues; is aware of the need to escalate problem resolution. - Can interact with state registries (electronic sick leave certificates, pension funds). - Can provide feedback outside the EHS. - Can use communication modules and technical support systems of various MIS. - Communicates in a timely manner regarding cases of infectious diseases and interacts to prevent epidemic outbreaks. - Performs online interaction in the provision of emergency medical care (creating medical records/dispatching/forming teams, and similar). - Interacts with Tsentral 103, Centers for Emergency Medical Care (CEMC), and similar. - Can interact with the information systems of the NHSU for processing contracts and agreements with healthcare facilities, with the Information System for Monitoring Socially Significant Diseases, with the Pension Fund of Ukraine, and similar. - Performs online interaction with a group of specialized databases and information systems (for example, "e-Stock," e-Krov, the embryological system, e-CPD, critical registries, and similar), as well as with niche professional systems at the local level. - Interacts with other organizations in Ukraine and with international information systems. |
| **Attitudes** | - Is aware of the need to escalate problems when their rapid resolution is not possible. - Treats participants of online interaction within eHealth with respect and tolerance. - Systematically provides feedback outside the EHS, especially in conditions of uncertainty, slowdowns, or excessive bureaucratization of work processes. |
| **PROFICIENCY LEVELS** | |
| **Level A (Basic)** | **At a basic level, with some autonomy following an instruction or template, or under the guidance of others, can interact:** with various information systems to address professional issues and to obtain information support for problems requiring advisory/consultative intervention by specialists; with a group of specialized databases and information systems of a specialized profile to receive/provide niche professional support and information. |
| **Level B (Sufficient)** | **At a sufficient level, independently, in line with one's own needs and solving clearly defined standard problems, can:** know how interaction with state registries (electronic sick leave certificates, pension funds) is carried out through centralized resources, such as the CDB of the EHS; provide feedback and exchange digital information within professional competence and in compliance with the requirements for the protection of personalized data outside the EHS at the horizontal level of interaction of eHealth components; use the communication models and technical support systems of various MIS in line with concluded contracts and the qualification requirements of the position of a healthcare worker; communicate in a timely manner regarding cases of infectious diseases and interact to prevent epidemic outbreaks; communicate, in the procedure established by law, from the user's workplace in the MIS and the EHS, with other specialized information systems for the provision/receipt of information from other electronic services in the procedure established by the Ministry of Health: emergency care; emergency medicine and disaster medicine; monitoring of socially significant diseases; stocks of medicines and medical products "e-Stock," e-Krov, and similar. |
| **Level C (Professional)** | **At a professional level, independently, in line with one's own needs and the needs of others, solving non-standard problems, can:** interact online in the provision of emergency medical care (creating medical records/dispatching/forming teams, and similar); interact with Tsentral 103, Centers for Emergency Medical Care (CEMC), and similar, using digital technologies of professional specialization and at an in-depth level; interact with the information systems of the NHSU for processing contracts and agreements with healthcare facilities, with the Information System for Monitoring Socially Significant Diseases, with the Pension Fund of Ukraine, and similar; perform online interaction with a group of specialized databases and information systems (for example, "e-Stock," e-Krov, the embryological system, e-CPD, critical registries, and similar), as well as with niche professional systems at the local level. |
| **Level D (High)** | **At a high professional or specialized level, including in complex contexts, can:** interact with other organizations in Ukraine and with international information systems on all critically important and urgent matters; recommend directions and ways of expanding interaction and communication with various information systems of eHealth for the broad implementation of digital technologies in healthcare. |
| **DOMAIN 4. DIGITAL HEALTH TOOLS, DEVICES, AND APPLICATIONS** | |
| **D4.C1. USE OF DIGITAL TOOLS IN MEDICAL (CLINICAL) PRACTICE (advanced diagnostics, screening, monitoring, treatment, rehabilitation, and similar in healthcare, by professional specialization)** | |
| **DESCRIPTOR** | |
| **Ability to use digital devices and tools for general laboratory and rapid diagnostics** in healthcare, as well as devices for functional analysis and general physiological measurements.  **Ensuring the reliability of diagnostic and monitoring results.** Compliance with requirements for the use of calibrated and certified digital devices and systems in practice. Critical and correct evaluation of rapid and laboratory test results; knowledge and understanding of the functional features of the digital tools used, their purpose, scope of application, and the influence of external factors on the process and methodology of investigations.  **Use of specialized devices and systems for advanced diagnostics** (digital X-ray, ultrasound, MRI, and similar). Application of filtering and image-processing methods to enhance visualization, contrast, and resolution. Signal extraction from noise; knowledge of methods for assessing the quality of clinical information in digital form.  **Use of screening methodologies,** by professional specialization; substantiation of investigation/experiment design and data collection methodology; digital processing of large datasets; digital support for forecasting and decision-making.  **Use of monitoring digital tools for long-term observation.** Collection, review, and analysis of clinical patient indicators; conduct of long-term monitoring studies during passive observation or during the provision of medical care, using stationary or mobile digital systems to verify preliminary results and build evidence-based medicine databases.  **Mastery of methods and technologies for applying modern digital tools and technologies** for emergency care, prevention and treatment of diseases, rehabilitation and restoration of functional status, and long-term patient care. | |
| **Knowledge** | - Knows modern methods, technologies, and instrumental (technical) tools for digital therapy and monitoring of the object of activity within the professional specialization in healthcare. - Knows the fields of application and limitations, advantages and disadvantages of these tools, depending on their technical parameters and characteristics and on the specifics of patient conditions. - Knows methods of determining the established values of monitored parameters, methods of ensuring their reliability, possible errors in the data obtained, and methods of eliminating them. |
| **Skills** | - Can use tools for digital therapy and monitoring of the object of activity in practice within the professional specialization in healthcare. - Can perform practical manipulations according to the relevant approved algorithms or improve them. - Can evaluate the results of digital investigations and therapeutic procedures, analyze data, and determine further treatment steps based on digital information. - Can interpret the results of digital therapy and/or monitoring of the object of activity and document them in line with the requirements accepted in the field. |
| **Attitudes** | - Recognizes the importance, new opportunities, and significance of digital diagnostics and therapy for emergency care, disease prevention and treatment, rehabilitation and restoration of functional status, and long-term patient care. - Takes responsibility for the reliability of conducting, documenting, and interpreting the results of diagnostic, screening, and monitoring investigations to build evidence-based medicine databases and conduct therapeutic and other treatment activities. |
| **PROFICIENCY LEVELS** | |
| **Level A (Basic)** | **At a basic level, with some autonomy following an instruction or template, or under the guidance of others, can:** perform simple template manipulations with digital therapeutic devices; document the results of digital therapy according to a given template, in line with standard requirements. |
| **Level B (Sufficient)** | **At a sufficient level, independently, in line with one's own needs and solving clearly defined standard problems, can:** verify the certification of digital therapeutic devices and the conformity of their characteristics with planned activities; configure and (as needed) calibrate digital therapeutic devices and prepare them for practical use; consciously, with an understanding of the expected outcome, use digital tools and technologies to perform technical manipulations; collect, review, and analyze clinical patient indicators using digital devices and technologies appropriate to the professional specialty and specialization; conduct long-term monitoring investigations during passive observation and/or during the provision of medical care using stationary or mobile digital systems. |
| **Level C (Professional)** | **At a professional level, independently, in line with one's own needs and the needs of others, solving non-standard problems, can:** professionally use specialized digital devices for advanced diagnostics, investigation, and long-term monitoring of the object of activity according to complex algorithms; substantiate the need for the use of digital tools and technologies in line with the complexity of tasks; apply advanced configurations of medical digital devices and systems and/or modern digital technologies for the additional processing of diagnostic information. |
| **Level D (High)** | **At a high professional or specialized level, including in complex contexts, can:** command methods and technologies for applying modern digital tools and technologies for emergency care, disease prevention and treatment, rehabilitation and restoration of functional status, and long-term patient care; use specialized digital devices and systems for advanced diagnostics (digital X-ray, ultrasound, MRI, and similar); ensure the reliability of digital diagnostic and long-term monitoring results; perform analysis, interpretation, and explanation of the results of digital diagnostics of the object of activity to build and improve evidence-based medicine databases. |
| **D4.C2. USE OF INTELLIGENT CLINICAL DECISION SUPPORT SYSTEMS (use of CDSS based on the intelligent analysis of clinical investigations, by professional specialization)** | |
| **DESCRIPTOR** | |
| **Ability to use advisory clinical decision support systems (CDSS).** Knowledge of the specifics of applying machine learning (ML) algorithms, artificial intelligence (AI), and big data analysis (BDA) for clinical decision support. Understanding of the opportunities and benefits of significantly reducing the risks of bias and subjectivity in diagnosis through the development and application of reliable ML and AI algorithms and reliable (verified) data. Knowledge of the digital toolkit of modern medical CDSS and full information about the necessity and appropriateness of their use in specific clinical cases. Risk assessment for specific patients. Awareness of the dependence of CDSS-recommended conclusions on the accuracy of the database/knowledge base and the correctness of the programmed system functioning. Ability to work with decision-making protocols (patient management based on data analysis — mathematical modeling and forecasting of patient status, intelligent systems/knowledge bases, and similar). Awareness that decisions based on the conclusions of artificial intelligence may not be entirely accurate. Critical attitude (identifying both positive and negative consequences) toward the use (collection, coding, and processing) of all data, especially personal data, by digital technologies driven by artificial intelligence. Understanding, evaluation, and readiness to assume full professional clinical responsibility for the results of the application of CDSS, AI, ML, and BDA. | |
| **Knowledge** | - Knows methods of formalizing healthcare tasks and their mathematical and computer modeling. - Knows and understands the basic principles and main approaches to implementing AI-based information CDSS. - Knows and understands strategies for obtaining medical knowledge using IT, and the principles of designing and using databases and knowledge bases of evidence-based medicine for implementing CDSS in healthcare and medical practice. - Knows and understands methods of forming logical conclusions based on knowledge and recommendations from experts and on data from evidence-based medicine, machine learning, and big data analysis. - Knows and understands the features, advantages, and disadvantages of applying medical CDSS in clinical practice and healthcare overall. - Has basic knowledge of modern methods and algorithms of artificial intelligence, machine learning, and data analysis used in CDSS. |
| **Skills** | - In line with the professional area, can apply digital CDSS methods and algorithms for the analysis and resolution of formalized healthcare tasks. - Can work with databases and knowledge bases and improve their structure. - Can build and use data samples for AI machine learning. |
| **Attitudes** | - Is willing to consider ethical issues related to artificial intelligence systems (for example, in contexts such as decision-making and the use of AI recommendations without human intervention). - Recognizes personal responsibility for the correctness of task formulation and the use of machine learning results, and for forming conclusions based on the application of AI CDSS. |
| **PROFICIENCY LEVELS** | |
| **Level A (Basic)** | **At a basic level, with some autonomy following an instruction or template, or under the guidance of others, can:** supplement and correct evidence-based medicine databases; update and organize expert conclusion data for the knowledge base in medical CDSS; use CDSS software products to obtain new recommendations for resolving tasks in treatment practice and forecasting the results of various activities in healthcare; analyze and assess the correctness of the obtained logical conclusions, taking into account the specific clinical case and the specific patient. |
| **Level B (Sufficient)** | **At a sufficient level, independently, in line with one's own professional needs (professional tasks/job duties), can solve clearly defined and standard problems:** based on comparative analysis, use and apply more reliable ML and AI algorithms and verified/improved databases and knowledge bases, taking into account the specifics of tasks addressed in the EHS and medical practice; build database samples for AI machine learning systems in line with the specifics of tasks addressed in the EHS and medical practice; assess risks and possible consequences of using insufficiently correct CDSS conclusions, inadequacy of AI and ML algorithms, and imperfections of BDA results. |
| **Level C (Professional)** | **At a professional level, independently, in line with one's own needs and the needs of others, solving non-standard problems, can:** apply in practice digital technologies and methods of deep learning, computer modeling, and big data analysis (BDA) implemented in the toolkit of CDSS within the EHS and its components; thoroughly command the digital toolkit of modern medical CDSS and full information about the necessity and appropriateness of their use in specific clinical cases or in the practical tasks addressed in healthcare; assume full professional clinical responsibility for the results of applying CDSS, AI, ML, and BDA. |
| **Level D (High)** | **At a high professional or specialized level, including in complex contexts, can:** use digital tools to improve and develop functional algorithms and software; improve CDSS for resolving complex tasks and problems in healthcare using AI, mathematical, and computer simulation modeling; substantiate new approaches to digital technologies for collecting, coding, and processing large medical datasets. |
| **D4.C3. USE OF INTEGRATED DIGITAL DEVICES AND APPLICATIONS (smart mobile and embedded digital devices, digital healthcare tools, intelligent sensors, patient manipulators, and similar)** | |
| **DESCRIPTOR** | |
| **Understanding and application of integrated digital technologies based on intelligent (smart) systems,** including embedded, mobile, distributed, and networked systems (mobile applications, telemedicine, Internet of Medical Things (IoMT), and similar) to improve diagnostics, treatment, therapy support, and patient care.  **Telemedicine / specialized care:** ability to determine and assess the necessity and appropriateness of the multidisciplinary use of telemedical hardware and software for providing consultative and diagnostic medical care, the remote performance of instrumental operations and manipulations, and the intelligent correction of the rehabilitation process; ability to take into account limitations and typical problems associated with remote medical care, in particular: patient suitability, monitoring of patient status and environmental conditions to prevent service-quality degradation, hardware capabilities, and the capacity and quality of communication and data transfer channels for making informed decisions; ability to analyze and evaluate the advantages of new methods and digital technologies compared with previous tools of the healthcare system; improvement of models and practices of their use in remote care delivery, with optimal application of digital technologies, human capacity, and consideration of the needs of healthcare professionals and patients.  **Intelligent digital devices:** ability to substantiate the conditions for the possibility, correctness, and appropriateness of using certified autonomous stationary and mobile smart devices and systems; practical command of working with them for monitoring and long-term observation of patients' vital functional indicators; ability to evaluate the correctness and appropriateness of using non-certified embedded and wearable devices — trackers, smartwatches, smart applications, and similar — for monitoring activity levels and other functional indicators of health, with the aim of improving patient care and monitoring patients' physical condition.  **Network technologies and IoT tools:** ability to apply in practice modern mobile medical digital devices and tools of the Internet of Medical Things, intelligent autonomous cyber-physical devices and systems for collecting data, accumulating information, analyzing it, and using it in medical practice and healthcare. Understanding and substantiation of the advantages and applications of cyber-physical biosensor systems for biomedical research, diagnostics, and other healthcare tasks. Understanding of the concept of and ability to use the Internet of Medical Things (IoMT): IoT devices, such as smart pills, smart inhalers, and smart patches, which can be used to monitor patient health, improve treatment adherence, and improve patient outcomes. Understanding and application of methods and means of data protection in IoT network technologies. | |
| **Knowledge** | - Knows and understands the specifics of applying modern digital technologies in embedded, mobile, distributed, and networked medical devices and systems and healthcare technical tools, and their functional advantages compared with traditional electronic systems used in healthcare (in line with the professional area). - Knows and understands the technical and technological specifics of applying digital telemedical hardware and software solutions for providing specialized care to patients, improving clinical practice, and addressing practical healthcare tasks (besides telecommunication and telemedical consultation tasks). - Knows and understands the advantages of using digital intelligent (smart) systems and technologies in healthcare, the methodology for evaluating the correctness of intelligent data processing results, and the possibilities of their practical use in healthcare tasks when using certified smart devices and systems and non-certified devices for express monitoring of patient vital parameters. - Knows the foundations of IoMT technology, the principles of functioning, and the practical use of its components for healthcare tasks. |
| **Skills** | - Can determine the necessary smart technologies and tools that are appropriate for use in addressing specialized problem-oriented healthcare tasks. - Uses modern digital tools, means, and technical solutions of telemedicine for providing specialized medical care to patients and addressing healthcare tasks. - Practically uses specialized smart systems and technologies in healthcare in line with the professional tasks being addressed. - Applies IoMT devices and tools in practice; performs information processing taking into account data protection in network, mobile, embedded, and distributed digital medical systems. |
| **Attitudes** | - Recognizes the advantages and necessity of mastering methods and ways of applying modern digital devices and intelligent computer technologies for resolving tasks and problems in healthcare. - Bears responsibility for the correct use of specialized digital devices and systems integrated into medical systems. - Understands the necessity of using certified digital systems in medical practice and for resolving a wide range of healthcare tasks. - Understands and substantiates the advantages and applications of robotic systems for disease prevention and patient rehabilitation in complex clinical cases, and of cyber-physical biosensor systems for biomedical research, diagnostics, and other healthcare tasks. |
| **PROFICIENCY LEVELS** | |
| **Level A (Basic)** | **At a basic level, with some autonomy following an instruction or template, or under the guidance of others, can:** assess the necessity and appropriateness of the specialized use of telemedical hardware and software complexes; remotely perform simple instrumental operations, manipulations, and rehabilitation process correction; apply certified intelligent autonomous stationary and mobile smart devices and systems and IoMT tools; apply practical skills in working with mobile smart devices and systems and IoMT tools. |
| **Level B (Sufficient)** | **At a sufficient level, independently, in line with one's own needs and solving clearly defined standard problems, can:** take into account limitations and typical problems associated with remote medical care; evaluate the correctness and appropriateness of using non-certified embedded and wearable devices — trackers, smartwatches, smart applications, and similar. |
| **Level C (Professional)** | **At a professional level, independently, in line with one's own needs and the needs of others, solving non-standard problems, can:** analyze and evaluate the advantages of new methods and technologies compared with traditional ones used in healthcare; improve models and practices for the use of specialized digital devices and systems in remote care delivery; at a high professional level, apply in practice modern mobile medical digital devices and tools of the Internet of Things, intelligent autonomous cyber-physical devices, and systems; improve methods and algorithms for the use of telemedicine, smart technologies, and IoMT. |
| **Level D (High)** | **At a high professional or specialized level, including in complex contexts, can:** optimize the application of digital telemedical tools, medical human capacity, and material resources; use components of the Internet of Medical Things (IoMT) ecosystem for monitoring patient health, improving treatment adherence, and improving expected patient outcomes; propose new approaches, methods, and algorithms for providing specialized care to patients, addressing current healthcare tasks, and investigating the effectiveness of applying embedded, mobile, distributed, and networked systems for diagnostics, treatment, rehabilitation, and patient care. |
| **D4.C4. APPLICATION OF INNOVATIVE DIGITAL TECHNOLOGIES IN HEALTHCARE (virtual (VR) and augmented (AR) reality, Internet of Medical Things (IoMT), 3D design and printing, CAD modeling, and similar; by professional specialization)** | |
| **DESCRIPTOR** | |
| **Open and informed attitude toward the introduction of innovative digital technologies in the workplace and in the healthcare system as a whole.** Investigation of trends and innovative solutions in the healthcare system; forecasting of progressive trends and competitive/non-competitive solutions in narrow professional specialization. Command and practical use of digital simulation modeling technologies in professional activities. Use of software applications for mathematical support and modeling of innovative technologies for diagnostics, monitoring, treatment, and disease prevention in healthcare. Knowledge of how to use virtual reality (VR) and augmented reality (AR) technologies in the treatment process, in line with the professional area. Ability to use medical technological digital (computerized) manipulators and robots for surgery and other medical manipulations, in line with the professional area. Ability to create CAD models and use 3D design and printing technologies in professional activities: in healthcare, for creating prostheses, implants, and surgical models, and 3D objects to assist in surgical planning. Awareness of the necessity of using technology for the safe implementation of digital innovative solutions in the healthcare system, including cryptography methods and blockchain technology. (Blockchain: blockchain technology is being explored for its ability to securely store and exchange data, in particular for medical records, research data, and clinical trials.) | |
| **Knowledge** | - Knows and understands the physical essence, theoretical foundations, and specifics of implementing innovative IT approaches: intelligent robots, virtual (VR) and augmented (AR) reality, 3D modeling, blockchain technology, and similar, for obtaining additional information about the object under study, providing it with new qualities, protecting data, and similar (with consideration of the professional specialization of the healthcare worker). - Knows and understands the features and existing limitations of a technical and ethical-legal nature regarding the application of digital innovations in healthcare. - Knows the foundations of computer-aided design (CAD systems), modeling, and its application in medicine for designing and optimizing medical devices and instruments. - Understands the principles of operation and application of the Internet of Medical Things (IoMT), which involves connecting medical devices and sensors to a network for collecting and exchanging data, contributing to improvements in diagnostics, monitoring, and patient treatment. - Understands the possibilities and limitations of using 3D design and printing in medicine, for example, for creating accurate replicas of organs for practical training, planning complex surgical interventions, or developing individual medical prostheses. |
| **Skills** | - Performs 3D modeling of the object of activity. - Uses virtual (VR) and augmented (AR) reality technologies in the treatment process, in planning surgical interventions, rehabilitation, and preventive activities, and similar. - Applies blockchain technology and crypto protection for secure storage and exchange of data in medical information systems. |
| **Attitudes** | - Recognizes the necessity and relevance of introducing innovative digital technologies in the workplace and in the healthcare system as a whole. - Strives to continuously update one's knowledge and understanding regarding the introduction of innovative digital technologies, progressive trends, and the competitiveness/non-competitiveness of new approaches and solutions in narrow professional specialization. - Shows interest in and willingness to introduce innovative digital technologies in the workplace and in the healthcare system as a whole. |
| **PROFICIENCY LEVELS** | |
| **Level A (Basic)** | **At a basic level, with some autonomy following an instruction or template, or under the guidance of others, can:** use available software to implement 3D models of the object of activity; implement simpler digital information protection protocols based on modern digital technologies of stream encryption, steganography, and blockchain; apply available software applications of virtual (VR) and augmented (AR) reality technologies. |
| **Level B (Sufficient)** | **At a sufficient level, independently, in line with one's own needs and solving clearly defined standard problems, can:** apply professional digital hardware and software products to address tasks of designing and practically implementing 3D structures of the object of professional activity; perform modeling of objects of professional activity according to standard algorithms for creating objects of virtual (VR) and augmented (AR) reality. |
| **Level C (Professional)** | **At a professional level, independently, in line with one's own needs and the needs of others, solving non-standard problems, can:** apply professional digital hardware and software products to create new models of 3D structures of the object of professional activity; address atypical innovative modeling tasks in healthcare; professionally develop new methods and algorithms for creating and practically using models of objects of virtual (VR) and augmented (AR) reality of objects of professional activity according to standard algorithms; assess risks of applying modern digital technologies and the reliability and correctness of their results. |
| **Level D (High)** | **At a high professional or specialized level, including in complex contexts, can:** practically use digital simulation modeling technologies in professional activities to improve innovative solutions and technologies in healthcare; use software applications and mathematical modeling methods to support innovative technologies for diagnostics, monitoring, treatment, disease prevention, and addressing other healthcare tasks; address issues of improving the protection of personalized data and professional information when using modern digital technologies in healthcare. |
| **D4.C5. PROFESSIONAL DEVELOPMENT AND RESEARCH WITH THE USE OF NEW DIGITAL TECHNOLOGIES (Research & Training, by professional specialization)** | |
| **DESCRIPTOR** | |
| Continuing professional development (CPD), practical research and applied investigations in healthcare, and innovative activity using new digital technologies.  **Lifelong learning and continuing professional development in the digital environment:** ability to use open digital educational resources (training, courses, educational programs, platforms) for one's professional and personal development anywhere and anytime. Improvement of professional practices, search for opportunities for self-development and continued learning, creation and development of one's own e-portfolio as a supplement to one's resume. Ability to direct one's own learning and the professional development of oneself and/or others in the healthcare system using digital technologies and tools.  **Continuous mastery of new digital technologies and improvement of skills in working with new digital tools.** Awareness of the necessity of continuous learning and adaptation to new digital technologies and developments in the medical field; improvement of skills; ability to select and use appropriate professional online courses (blended or distance learning). Ability to effectively use digital technologies and tools for self-learning and teaching others; for organizing the educational process in specialized and professional educational institutions of the healthcare system.  **Research and innovation activity:** interest in using digital technologies in scientific work and professional activity. Capacity for creative thinking, scientific research, and clinical research work using modern digital technologies and tools. Ability to set and address tasks that require a creative, innovative approach using digital technologies. Ability to plan, optimize, and conduct experiments and long-term studies in healthcare. Command and practical use of modern software packages and digital applications for the statistical processing of experimental results and their presentation; correct and evidence-based formulation of conclusions and recommendations regarding their practical use. Ability to contribute to applied research programs on the application of digital technologies in healthcare (in line with the professional specialization). | |
| **Knowledge** | - Knows new digital technologies for learning and research and the specifics of their application in medicine and healthcare. - Knows modern digital platforms for organizing the educational process in various formats (in-person, distance, blended). - Knows the requirements regarding the specifics of presenting and formatting educational materials of digital training courses for their effective mastery. - Knows the technical capabilities for applying new hardware and software solutions of digital information technologies and systems for conducting research-intensive investigations and developments in healthcare. - Knows the capabilities of modern software for collecting, processing, interpreting, modeling, and presenting the results of scientific research in healthcare. - Knows the principles and practices of digital ethics and data confidentiality in the context of scientific research and the medical field. - Knows methods of data validation and assessment of the quality of research data in the digital environment. |
| **Skills** | - Can work with search engines and web resources of professional, including narrow professional (by specialization), focus to ensure ongoing professional development. - Can effectively use digital technologies and tools for self-learning and teaching others. - Can organize the educational process in specialized institutions of professional pre-higher, higher, and postgraduate education in the healthcare system in in-person and/or distance modes (medical staff, physicians, and other healthcare facility workers). - Can set and address scientific tasks that require a creative, innovative approach and the application of modern digital technologies. - Can substantiate the choice of digital technology/platform for planning, optimizing, and conducting experiments and long-term studies in healthcare. - Commands and practically uses modern software packages and digital applications for modeling, statistical processing of experimental results, and their presentation; correctly and with evidence formulates conclusions and recommendations regarding their practical use. |
| **Attitudes** | - Recognizes the vital necessity of constantly learning and adapting to new digital technologies and developments in the medical field. - Is interested in using digital technologies in scientific work and creativity. - Has an internal need for communication and discussion of professional matters related to research or innovation activity. - Has the capacity for and interest in creative activity, the search for new facts and patterns, the construction of logical interconnections among them, and the inclination to apply IT to explain them. - Has an inclination toward self-organization and the planning of such activity. - Has the capacity for generalization, logical thinking, formulation of rules and statements, and modeling of situations. - Welcomes the challenges and opportunities offered by digital technologies for improving the quality of education, healthcare, and scientific research. |
| **PROFICIENCY LEVELS** | |
| **Level A (Basic)** | **At a basic level, with some autonomy following an instruction or template, or under the guidance of others, can:** search for current new educational and professional information using IT technologies and resources; pursue professional self-development through relevant professional online/offline courses; conduct scientific search and/or simple research activities using IT technologies on a defined professional topic and a simple research plan; at the minimum required level, use modern software packages and digital applications for modeling, statistical processing of experimental results, and their presentation. |
| **Level B (Sufficient)** | **At a sufficient level, independently, in line with one's own professional needs (professional tasks/job duties), can solve clearly defined and standard problems:** evaluate the relevance and quality of services and the level of trust in educational platforms, courses, and resources; pursue professional self-development by selecting higher-quality content of relevant professional online/offline courses (analysis of content and form of educational material presentation); conduct training seminars in a research group using typical digital training platforms/technologies; substantiate the formulation of simple tasks and the conduct of standard investigations requiring a creative, innovative approach, using modern digital devices, manufacturer-certified software, and standard software packages. |
| **Level C (Professional)** | **At a professional level, independently, in line with one's own needs and the needs of others, solving non-standard problems, can:** continuously update professional knowledge and pursue professional development by mastering new digital technologies and developments in the medical field; use digital technologies and tools for self-learning and teaching others; apply modern digital technologies and tools in scientific search and clinical research work; substantiate, set, plan, and address non-standard research tasks that require a creative, innovative approach and the application of the latest achievements in computer technologies; plan, optimize, and conduct complex scientific experiments and long-term investigations in healthcare; professionally command and practically use modern software packages and applied digital applications; create and develop digital training materials that meet the needs of educational process participants. |
| **Level D (High)** | **At a high professional or specialized level, including in complex contexts, can:** highly effectively use digital technologies and tools for self-learning and teaching others; apply new digital technologies and tools and innovative approaches in research and practical activities within the EHS ecosystem, in setting and addressing professional tasks; plan, optimize, and conduct experiments and long-term monitoring investigations in healthcare using mobile, embedded, and distributed digital specialized systems; professionally use modern software packages and digital applications for the statistical processing and modeling of experimental results, applying them to correctly and with evidence formulate conclusions and recommendations regarding their practical use; contribute one's own proposals/methodologies/improvements to the algorithms of one's department's research program and propose effective improvements to the hardware-software solutions of digital systems to enhance their functioning and research outcomes. |
| **DOMAIN 5. DIGITAL HEALTH TRANSFORMATION AND LEADERSHIP** | |
| **D5.C1. DIGITAL LEADERSHIP AND MANAGEMENT OF DIGITAL TRANSFORMATIONS** | |
| **DESCRIPTOR** | |
| **Digital leadership in healthcare** means the use of digital technologies to improve the processes of medical/non-medical service delivery and operational efficiency at the organizational level. This concerns leaders of healthcare facilities, heads of major healthcare units, heads of operational units in support services whose activities take place in the field of medical care delivery, and other healthcare leaders. Heads of enterprises, institutions, facilities, and organizations of healthcare at various levels must ensure effective digital transformation in healthcare at the relevant level, which includes: the ability to determine the level of digital readiness of the facility/institution/unit; the ability to determine the needs and requirements of the healthcare facility for managing digital transformation processes in line with: specific patient categories (taking into account demographic features, clinical needs, preferences); the local working environment (systems, staffing structure, leadership, and similar); and the specific needs and preferences of the staff of the facility/department or other unit (including analysis of digital competence gaps in the workforce); the ability to think strategically: the ability to develop a clear vision and strategy for digital transformation that aligns with the overall goals and objectives of the organization/institution/facility; the ability to develop digital transformation action plans that align with the overall goals and objectives of the organization, institution, region, and country; the ability to build teams of specialists in digital technologies and to foster a culture of digital innovation and experimentation among employees; supporting the improvement of digital literacy in the healthcare system; supporting the development of e–public health; the ability to develop and implement a multisectoral interaction and population information scheme in healthcare emergencies using digital technologies. At the sectoral level/at the level of leaders of medical educational institutions, has the ability to ensure: the training of teachers in numbers sufficient to launch the implementation of the Digital Competence Framework at all levels and forms of education; the preparation and dynamic updating of curricula for each level of education. | |
| **Knowledge** | - Knows digital leadership and digital transformation in healthcare. - Knows the digital readiness index of an educational institution. - Knows strategy/strategic action plan/digital transformation programs. - Knows the criteria for evaluating the effectiveness of facility digitalization. - Knows how to audit existing information systems and registries in the field within one's competencies. - Knows digital infrastructure and digital initiatives in healthcare. - Knows MIS, EHS, electronic registries and classifiers, digital competence, digital literacy, and e–public health. - Knows how to determine the state of digital readiness of healthcare facilities. |
| **Skills** | - Can ensure effective digital transformation in healthcare at the relevant level. - Can determine the needs and requirements of the healthcare facility for planning and managing the strategic digital transformation program in line with: specific patient categories (taking into account demographic features, clinical needs, preferences); the local working environment (systems, staffing structure, leadership, and similar); the specific needs and preferences of department staff, including digital competence gaps among workers. - Can develop digital transformation strategies and action plans that align with the overall goals and objectives of healthcare enterprises, institutions, facilities, and/or organizations. - Can build teams of specialists in digital technologies and foster a culture of digital innovation and experimentation among employees. - Can effectively manage the change process during digital transformation in healthcare. - Can evaluate the state of digital transformation, which digital solutions and initiatives have already been implemented in the facility or healthcare system, and assess their effectiveness and alignment with needs and goals. |
| **Attitudes** | - Approaches digital transformations in healthcare responsibly. - Weighs the advantages and disadvantages of using digital technologies in healthcare. - Creates an atmosphere of readiness and openness to the implementation of digital innovations among employees. - Demonstrates readiness for cooperation and partnership with other organizations, technology providers, and stakeholders for the joint development and implementation of digital initiatives in healthcare. - Expresses concern that many online services may not be accessible to all. - Develops eHealth, including e–public health, at the relevant level appropriately. - Has the capacity to adapt to a rapidly changing digital environment and adopt new ideas and innovations in healthcare. |
| **PROFICIENCY LEVELS** | |
| **Level A (Basic)** | **At a basic level, with some autonomy following an instruction or template, or under the guidance of more experienced specialists, knows how to:** identify needs and develop a simple action plan for the digital transformation of a facility; create and manage teams of digital technology specialists to achieve defined goals. |
| **Level B (Sufficient)** | **At a sufficient level, independently, in line with job duties and operational needs, solving clearly defined standard problems, knows how to:** evaluate the state of digital readiness of a healthcare facility; identify problems and possibilities for resolving them through digital technologies; develop and implement an action plan for the digital transformation of the facility/institution/organization; build a team of digital technology specialists to achieve the goals defined in the action plan. |
| **Level C (Professional)** | **At a professional level, independently, in line with the professional needs of the facility/institution/organization, solving non-standard problems, can:** periodically analyze the state of digital readiness of the healthcare facility; systematically and consistently carry out the digital transformation of the facility/institution/organization; ensure effective digital transformation in healthcare. |
| **Level D (High)** | **At a high professional or specialized level, including in complex contexts, knows how to:** systematically analyze the state of digital readiness and development of healthcare facilities; develop/update the strategic vision and/or strategy of digital transformation of the facility/institution/organization in line with challenges, taking into account the emergence of new technological solutions and opportunities in healthcare; achieve significant goals in improving healthcare facility management processes, improving work processes, and achieving better outcomes through digital transformation in healthcare facilities. |
| **D5.C2. AUTOMATION OF MANAGEMENT AND TECHNOLOGICAL SUPPORT OF WORK PROCESSES IN HEALTHCARE FACILITIES (use of digital technologies for the automation of work processes — technological support and management of processes in healthcare facilities)** | |
| **DESCRIPTOR** | |
| **Healthcare leaders must possess the following competencies to ensure the automation and organizational support of work processes through digital technologies in healthcare facilities, in particular:** ability to recognize and implement new digital technologies and digital infrastructure for the digitalization of business processes; capacity to make informed decisions on the automation of work processes that positively affect the efficiency of the organization/institution/facility; capacity to address technical and professional problems through digital technologies; use of digital technologies for business analytics, the calculation of medical service tariffs and payment processing, contracting, reporting and processing of statistical data, monitoring, and the management of logistics and warehouse balances, and similar; change management: the ability to manage organizational changes, including the implementation of new digital technologies for improving business processes; capacity to collect, analyze, and interpret large volumes of data and use them for administrative decision-making and the improvement of work processes; ability to use specialized project management software: the capacity to plan, organize, and manage digital projects, including coordination with cross-functional teams and ensuring on-time and on-budget project delivery; ability to select the right digital tools and means depending on the specific needs and goals of the organization, conducting research and thoroughly evaluating options before making a decision. | |
| **Knowledge** | - Knows the existence of digital tools for optimizing and automating work processes, contracting, reporting and processing of statistical data, monitoring, and the management of logistics and warehouse balances, and similar. - Knows the availability of digital tools for supporting administrative decision-making and specialized project management software, including coordination with cross-functional teams and ensuring on-time and on-budget project delivery. |
| **Skills** | - Can recognize and implement new digital technologies and digital infrastructure for the digitalization of business processes. - Uses digital technologies for the calculation of medical service tariffs and payment processing, contracting, reporting and processing of statistical data, monitoring, and the management of logistics and warehouse balances, and similar. - Can implement Electronic Medical Records (EMR) systems in healthcare facilities. - Knows how to collect, analyze, and interpret large volumes of data for administrative decision-making and the improvement of work processes. - Knows how to use specialized project management software: planning, implementation, and evaluation of the effectiveness of work projects, including coordination with cross-functional teams and ensuring on-time and on-budget project delivery. - Can select the right digital tools and means depending on the specific needs and goals of the organization, conducting research and thoroughly evaluating options before making a decision. - As needed, has the capacity to develop and present training materials and conduct trainings for stakeholders to prepare and train them in the use of updates, digital tools, and systems. |
| **Attitudes** | - Makes informed, substantiated decisions on the automation of work processes that positively affect the efficiency of the organization/institution/facility. - Is ready for new technological challenges and manages organizational changes, including the implementation of new digital technologies for improving business processes. - Is motivated to achieve the improvement of work processes and organizational efficiency through the implementation of digital technologies and automation. |
| **PROFICIENCY LEVELS** | |
| **Level A (Basic)** | **At a basic level, with some autonomy following an instruction or template, or under the guidance of others, knows how to:** recognize and implement new digital technologies and digital infrastructure for the digitalization of business processes; automate individual work processes. |
| **Level B (Sufficient)** | **At a sufficient level, independently, in line with job duties and professional needs, solving clearly defined standard problems, knows how to:** select the right digital tools and means depending on specific needs; automate a significant share of business processes. |
| **Level C (Professional)** | **At a professional level, independently, in line with organizational needs and the needs of healthcare facility workers, solving non-standard problems, knows how to:** automate business processes in healthcare facilities, listening to the operational needs of healthcare workers; use specialized project management software, including coordination with cross-functional teams and ensuring on-time and on-budget project delivery. |
| **Level D (High)** | **At a high professional or specialized level, including in complex contexts, knows how to:** implement new digital technologies and digital infrastructure for the automation of a greater number of business processes; collect, analyze, and interpret big data for administrative decision-making and the improvement of work processes; select the right digital tools and means depending on the specific needs and goals of the organization, conducting research and thoroughly evaluating options before making a decision. |
| **D5.C3. TECHNOLOGICAL SUPPORT OF PERSONNEL MANAGEMENT** | |
| **DESCRIPTOR** | |
| **Strengthening the workforce capacity and improving labor efficiency through the use of electronic personnel accounting systems, electronic personnel reporting, registries, work schedules, payroll software, and personnel motivation, and similar.** Healthcare leaders need a range of digital skills to optimize personnel management processes within the framework of digital transformation in healthcare, in particular: knowledge of how to use personnel management systems that automate and optimize processes such as recruitment, onboarding, performance management, and conformity to qualification requirements; the ability to implement personnel performance management systems and work efficiency dashboards; to set goals and objectives; and to provide feedback and recognition. Capacity to select the right tool depending on the specific needs and goals of the organization; conducting research and thoroughly evaluating options before making a decision. Capacity to create appropriate working conditions, in particular digital workplaces for healthcare workers. Ability to communicate and collaborate with staff and patients in the digital environment. For leaders, to improve personnel management processes in the digital environment, it is also important to have the ability to analyze and interpret big data for decisions related to personnel management; to communicate and collaborate effectively in the digital environment with various stakeholders, including employees, and to work with them to achieve common goals. Ability to manage projects through digital technologies: planning, organizing, and managing digital projects related to personnel management, including coordination with cross-functional teams and ensuring on-time and on-budget project delivery. Ability to manage change: the capacity to manage organizational changes, including the implementation of new technologies and processes related to people management. Knowledge of how to assess the level of digital competence required of staff and how to develop them to support digital transformation processes at the organizational level. Knowledge of the foundations of implementing digital learning management systems (LMS) and personnel development programs through digital technologies (CPD). Capacity to provide continuous training for staff and to support their willingness to continuously learn and adapt to new technologies and developments in people management, including through the use of digital technologies. Ability to build relationships with key stakeholders for the digital transformation of the facility within the development of the digital ecosystem of healthcare. | |
| **Knowledge** | - Knows personnel management systems capable of automating and optimizing processes such as recruitment, onboarding, performance management, and conformity to qualification requirements; implementing personnel performance management systems and work efficiency dashboards; setting goals and objectives; and providing feedback and recognition. - Knows the necessity of creating appropriate working conditions, in particular digital workplaces for healthcare workers. - Knows the necessity of assessing and developing the digital competence of workers and the existence of digital online learning management systems. - Understands the principles of digital marketing and its use to attract and retain personnel and to improve the reputation of the facility. - Knows applications for managing payroll, work schedules, and other personnel-related aspects; for managing the recruitment process, documentation, electronic signatures, and other personnel-related aspects; for personnel management, performance evaluation, training and development; and for managing work schedules and tracking working hours. |
| **Skills** | - Can automate and improve personnel management processes within the framework of digital transformation in healthcare, in particular: implement Human Resource Management (HRM) systems, electronic personnel accounting systems, electronic personnel reporting, registries, work schedules (Workforce Management — WFM), payroll software, and personnel motivation. - Can manage personnel performance and implement work efficiency dashboards. - Can create appropriate working conditions, in particular digital workplaces for healthcare workers, and apply mobile applications that allow employees to access information about their work, monitor work schedules, and communicate with colleagues anytime and from anywhere. - Can analyze and interpret digital data for decisions related to personnel management. - Can communicate and interact effectively with personnel through digital tools and communication platforms. - For leaders, to improve personnel management processes in the digital environment, it is also important to communicate and collaborate effectively in the digital environment with various stakeholders, including employees, and to work with them to achieve common goals. - Uses video monitoring systems that allow tracking of personnel work, ensuring workplace safety, and assisting in resolving conflict situations. - Creates conditions for providing feedback in digital format. - Knows how to assess the level of digital competence required of workers and how to develop them in the digital environment. - Knows how to implement digital online learning management systems and development programs (CPD), using Learning Management Systems (LMS) that allow healthcare facilities to organize distance learning for personnel, assess the knowledge and skills of employees, and maintain records of completed training and certifications. - Can evaluate and analyze the results of digital personnel management initiatives to ensure continuous improvement and process optimization. |
| **Attitudes** | - Selects the right digital tools depending on the specific needs and goals of the organization, conducting research and thoroughly evaluating options before making a decision. - Cares about improving the digital literacy of workers, ensures continuous training, and helps them adapt to new digital technologies. |
| **PROFICIENCY LEVELS** | |
| **Level A (Basic)** | **At a basic level, with some autonomy following an instruction, or under the guidance of others, knows how to:** implement individual digital solutions for personnel management; establish digital communication; verify the level of digital literacy of healthcare facility workers. |
| **Level B (Sufficient)** | **At a sufficient level, independently, in line with job duties and operational needs, solving clearly defined standard problems, knows how to:** communicate and collaborate effectively in the digital environment with various stakeholders, including employees, and work with them to achieve common goals; manage organizational changes, including the implementation of new technologies related to personnel management; implement automated personnel management systems; ensure continuing professional development of personnel, including in distance or blended learning formats. |
| **Level C (Professional)** | **At a professional level, independently, in line with the needs of the facility/organization and the needs of workers, solving non-standard problems, can:** manage organizational changes, including the implementation of new technologies and processes related to people management; select the right digital tools depending on the specific needs and goals of the organization, conducting research and thoroughly evaluating options before making decisions on the automation of personnel management. |
| **Level D (High)** | **At a high professional or specialized level, including in complex contexts, can:** highly professionally ensure the automation of personnel management processes, taking into account the needs of workers, the challenges of remote work, and wartime; develop and/or implement effective digital systems and tools for personnel management. |
| **D5.C4. MANAGEMENT OF OBJECTS, RESOURCES, AND ASSETS OF eHEALTH. BUSINESS ANALYTICS (resource management technologies; systems analysis of operations and management to enable the sustainable development of healthcare facilities)** | |
| **DESCRIPTOR** | |
| **Capacity to implement automated management systems. Systems analysis and big data.** Capacity to analyze data and make decisions based on digital tools and data analysis; use digital technologies for business analytics and distinguish problems for which the application of systems analysis is appropriate; capacity to interpret the basic concepts of systems analysis and the principles of a systems approach to computer information systems and objects of computerization; capacity to classify systems and methods of systems modeling and apply analytical and synthetic approaches to modeling information systems; apply methodologies, methods, and algorithms of systems analysis to address problems on complex objects of computerization; capacity to audit existing information systems and registries in the field within one's competencies; knowledge of how to invest in the digital technologies and infrastructure required by a facility/institution to support digital transformations; ability to assess the advantages and disadvantages of various MIS for the needs of the healthcare facility and to substantiate the optimal choice; ability to build a system of indicators for evaluating and monitoring effectiveness depending on needs and goals. | |
| **Knowledge** | - Knows automated management systems, systems analysis, data, big data, computer information systems; methods of systems modeling and systems analysis; eHealth, technical issues of the EHS, protection of patient information, new technologies and developments in eHealth, regulatory acts, HIPAA. - Knows the existence of digital tools for business analytics. - Knows and understands the relevant data metrics for measuring the effectiveness of healthcare system indicators. - Knows the main regulatory acts and standards regarding eHealth (for example, HIPAA) and how to ensure compliance with them. - Knows about the analysis of healthcare system indicators and quantitative strategies, in particular: industrial indicators of the quality of medical care delivery — Pareto efficiency criteria, Taguchi loss criteria, Weibull distribution criteria, and others. |
| **Skills** | - Can distinguish problems for which the application of systems analysis is appropriate. - Can interpret the results of systems analysis. - Can classify systems and methods of systems modeling; apply analytical and synthetic approaches to modeling information systems. - Can apply methods and algorithms of systems analysis to address problems on complex objects of computerization. - Manages change and avoids errors and risks; has the capacity to collect, analyze, and interpret large volumes of data and use them for management decision-making. - Performs technical troubleshooting: capacity to diagnose and resolve technical problems related to eHealth. - Uses digital technologies to optimize medical and financial resources and human capacity for the needs of patients and the healthcare system at various levels, in particular ERP (Enterprise Resource Planning), CRM (Customer Relationship Management), WMS (Warehouse Management System), and other information system packages. - For effective management of eHealth systems and the minimization/avoidance of errors and risks, healthcare workers need a range of digital skills, in particular: technical expertise — knowledge of various eHealth systems and available tools, such as electronic health records, and the capacity to understand and apply them in their work processes; data entry and management — capacity to enter and manage patient data accurately and efficiently in eHealth; data analysis and interpretation — capacity to collect, analyze, and interpret large volumes of data and use them for decision-making and improving patient care; technical troubleshooting — capacity to diagnose and resolve technical problems related to eHealth; cybersecurity awareness — knowledge of potential cybersecurity risks related to eHealth and capacity to follow best practices for protecting patient data; use of specialized secure communication channels; continuous learning — capacity and willingness to continuously learn and adapt to new technologies and developments in eHealth; compliance with regulatory acts — knowledge of regulatory acts applicable to eHealth, such as the Law of Ukraine "On the Protection of Personal Data," and capacity to ensure compliance with them. |
| **Attitudes** | - Approaches compliance with regulatory acts and standards regarding eHealth responsibly. - Strives to use digital technologies to optimize resource management. |
| **PROFICIENCY LEVELS** | |
| **Level A (Basic)** | **At a basic level, with some autonomy following an instruction or template, or under the guidance of others:** performs systems analysis of large operational data; conducts an audit of existing information systems and registries in the field within one's competencies. |
| **Level B (Sufficient)** | **At a sufficient level, independently, in line with one's own needs and solving clearly defined standard problems, can:** analyze data and make decisions based on digital tools and data analysis; assess the advantages and disadvantages of various MIS for the needs of the healthcare facility and substantiate the optimal choice. |
| **Level C (Professional)** | **At a professional level, independently, in line with one's own needs and the needs of others, solving non-standard problems, can:** use digital technologies for business analytics and distinguish problems for which the application of systems analysis is appropriate; interpret the basic concepts of systems analysis and the principles of a systems approach to computer information systems and objects of computerization. |
| **Level D (High)** | **At a high professional or specialized level, including in complex contexts, can:** implement automated management systems; classify systems and methods of systems modeling; apply analytical and synthetic approaches to modeling information systems; apply methodologies, methods, and algorithms of systems analysis to address problems on complex objects of computerization; know how to invest in the digital technologies and infrastructure required by a facility/institution to support digital transformations. |
| **D5.C5. RISK ASSESSMENT AND PROBLEM-SOLVING IN THE DIGITAL ENVIRONMENT** | |
| **DESCRIPTOR** | |
| **Risk assessment is an important step in any digital transformation initiative in healthcare,** as it helps leaders identify and prioritize potential risks and develop strategies for their mitigation, minimization, or avoidance. It is important to have a regular and consistent risk assessment process, as technologies and the environment are constantly changing. Ability to assess and manage cybersecurity risks (since the more confidential patient information is stored and transmitted in digital form, the higher the risk of data leaks and cyberattacks). At the organizational level, knowledge of how to avoid risks regarding the preservation of information confidentiality (digitalization involves the collection and exchange of large volumes of personal and confidential information, which may raise concerns about confidentiality and data protection). Awareness of the possibility of creating situations of technical dependence at the organizational level on computer technologies, which can be problematic in the event of system failures, power outages, or lack of internet connection. Awareness that digitalization can amplify existing inequalities in access to medical care, especially for marginalized communities that may lack access to technology and have low levels of digital literacy. Ability to create reliable security and confidentiality protocols and invest in regular security and vulnerability assessments and penetration testing to ensure the protection of confidential data. Engagement of a wide range of stakeholders, including healthcare providers, employees, patients, and policymakers, to ensure that all viewpoints are taken into account. | |
| **Knowledge** | - Has cybersecurity awareness: knowledge of potential cybersecurity risks related to eHealth and capacity to follow best practices for protecting patient information; cyberattack, cybersecurity; personal and confidential information; financial information; patient data leaks, big data, confidentiality and data protection, security and confidentiality protocols, vulnerabilities and penetration testing. - Knows protection from unauthorized access, use, disclosure, breach, modification, or destruction of data. - Knows protection from cyber threats, such as hacking, malware, and phishing attacks, and ransomware attacks (malware that encrypts patient data and demands payment to restore access), which may disrupt the operation of the healthcare system. - Knows the requirements for compliance with laws and regulations related to the protection of patient data. - Knows the strategic plan of digital transformation, strategies for the minimization or avoidance of ethical risks, cyber risks, and other risks of digital transformations; the development and implementation of training materials and measures regarding rules of personnel conduct in cyberspace; technical dependence of the organization/institution; inequality in access to digital medical care. - Knows the vulnerability of medical devices (IoMT) such as pacemakers and insulin pumps, which may be vulnerable to hacking and other cyber threats, with potentially serious consequences for patient safety. - Knows phishing attacks: attempts to deceive users into disclosing confidential information, such as login credentials or personal information, which may lead to data leaks and other security incidents. - Knows about compliance violations: failure to comply with laws and regulations regarding the protection of patient data, such as HIPAA, may result in fines and other sanctions. - Knows about disruptions of healthcare system operations: cyberattacks may disrupt the delivery of healthcare services, causing delays and other operational problems. - Knows about reputational damage: cybersecurity incidents may damage the reputation of a medical organization, leading to a loss of trust and patients. |
| **Skills** | - Can assess and minimize the risks of digital transformations. - Can identify and prioritize potential risks. - Can develop strategies and strategic action plans for the minimization or avoidance of cyber risks. - Can manage cybersecurity risks. - Knows how to avoid risks regarding the preservation of information confidentiality. - Is aware of the possibility of creating situations of technical dependence at the organizational level. - Is aware that digitalization can amplify existing inequalities in access to medical care, especially for marginalized communities that may lack access to technology and have low levels of digital literacy. - Can create reliable security and confidentiality protocols and invest in regular security and vulnerability assessments and penetration testing. - Knows how to use digital technologies to address problems. - Can identify and resolve problems related to eHealth. - Can adapt and improve digital transformation plans in line with unforeseen events or external influences. - Performs technical troubleshooting and problem-solving in the digital environment: capacity to identify and resolve problems related to eHealth, as well as find solutions; capacity to adapt and improve digital transformation plans in line with unforeseen events or external influences; capacity to address technical and professional problems through digital technologies; capacity to perform continuous monitoring and evaluation of the impacts and effectiveness of digital initiatives, and use data to inform decision-making and further improvement; capacity to identify problems that arise during the implementation of technologies and automation and to develop effective strategies for resolving them. |
| **Attitudes** | - Approaches risk assessment carefully, including cyber threats and other risks in the digital environment of healthcare facilities. - Systematically, consistently, and responsibly implements reliable cybersecurity measures, such as firewalls, intrusion detection systems, encryption, and security training for employees. - Does not neglect regular security assessments and incident response planning, which are important for identifying vulnerabilities and managing security incidents. |
| **PROFICIENCY LEVELS** | |
| **Level A (Basic)** | **At a basic level, with some autonomy following an instruction or template, or under the guidance of others:** knows about the existence of problems related to eHealth; knows about the existence of risks of digital transformations; knows that plans for minimizing cyber risks exist; knows how to preserve confidential information at the organizational level; is aware of the possibility of situations of technical dependence in the absence of the internet, electricity, and similar. |
| **Level B (Sufficient)** | **At a sufficient level, independently, in line with professional tasks/job duties and one's own professional needs, solving clearly defined and standard problems:** can assess and minimize the risks of digital transformations; knows the necessity of conducting regular cybersecurity and vulnerability assessments and penetration testing; together with cybersecurity specialists, can develop action plans for the minimization of cyber risks, directing cybersecurity specialists; manages cybersecurity risks; knows how to avoid risks regarding the preservation of information confidentiality; knows how to avoid situations of technical dependence at the organizational level (availability of alternative power sources, multiple levels of information protection, and similar); knows the necessity of creating reliable security and confidentiality protocols, vulnerability assessments, and penetration testing; at the organizational level, can identify and resolve problems related to eHealth. |
| **Level C (Professional)** | **At a professional level, independently, in line with one's own needs and the needs of others, solving non-standard problems, can:** develop strategies for the minimization and avoidance of cyber risks, manage cybersecurity risks, and ensure the preservation of information confidentiality; anticipate and develop measures to avoid situations of technical dependence at the organizational level; minimize inequality in access to medical care, especially for marginalized communities that may lack access to technology and have low levels of digital literacy; can create reliable security and confidentiality protocols and invest in regular security and vulnerability assessments and penetration testing; knows how to use digital technologies to address problems; identify and resolve problems related to eHealth; adapt and improve digital transformation plans in line with unforeseen events or external influences. |
| **Level D (High)** | **At a high professional or specialized level, including in complex contexts, can:** systematically assess, prioritize, and avoid risks of digital transformations at the organizational level; at the organizational level, confidently and skillfully create strategies for the avoidance of cyber risks; manage cybersecurity risks; know how to ensure the preservation of information confidentiality at the organizational level; have technological solutions to avoid situations of technical dependence at the organizational level; be aware that digitalization can amplify existing inequalities in access to medical care, especially for marginalized communities that may lack access to technology and have low levels of digital literacy; create reliable security and confidentiality protocols and invest in regular security and vulnerability assessments and periodic penetration testing; use digital technologies to address problems; identify and resolve problems related to eHealth; adapt and improve digital transformation plans in line with unforeseen events or external influences. |
